# Soret and Dufour effects on MHD peristaltic transport of Jeffrey fluid in a curved channel with convective boundary conditions

**DOI:** 10.1371/journal.pone.0164854

**Published:** 2017-02-21

**Authors:** Tasawar Hayat, Hina Zahir, Anum Tanveer, Ahmad Alsaedi

**Affiliations:** 1 Department of Mathematics, Quaid-I-Azam University 45320, Islamabad 44000, Pakistan; 2 Department of Electrical and Computer Engineering, Faculty of Engineering, King Abdulaziz University, Jeddah 21589, Saudi Arabia; Worcester Polytechnic Institute, UNITED STATES

## Abstract

The purpose of present article is to examine the peristaltic flow of Jeffrey fluid in a curved channel. An electrically conducting fluid in the presence of radial applied magnetic field is considered. Analysis of heat and mass transfer is carried out. More generalized realistic constraints namely the convective conditions are utilized. Soret and Dufour effects are retained. Problems formulation is given for long wavelength and low Reynolds number assumptions. The expressions of velocity, temperature, heat transfer coefficient, concentration and stream function are computed. Effects of emerging parameters arising in solutions are analyzed in detail. It is found that velocity is not symmetric about centreline for curvature parameter. Also maximum velocity decreases with an increase in the strength of magnetic field. Further it is noticed that Soret and Dufour numbers have opposite behavior for temperature and concentration.

## Introduction

Undoubtedly the peristalsis of fluids in a channel is useful in several applications in engineering and biomechanics. The importance of topic can be recognized by its numerous physiological and industrial applications about swallowing food through esophagus, capillaries and arterioles, in the vasomotion of venules, in sanitary fluid transportation, toxic liquid transport in the nuclear industry, locomotion of worms, roller and finger pumps etc. Peristalsis is a radially symmetrical contraction and relaxation of muscles so as to propagate in a wave down a tube, in an anterograde way. In digestive tract (for instance the human gastrointestinal tract) a peristaltic wave is produced due to smooth muscle tissue contraction which forces a ball of food (entitled a bolus as in the upper gastrointestinal tract, in the esophagus and chyme in the stomach) across the tract. A non-Newtonian fluid model for the study of peristasis in a non-uniform rectangular duct has been investigated by Ellahi et. al [1]. Another useful article, peristaltic flow with thermal conductivity of water with copper nanofluid is done by Akbar et. al [2]. Peristaltic transport of magnetohydrodynamic (MHD) physiological fluids are important in medicine and bioengineering. In fact electric current is induced due to movement of conducting liquid across the magnetic field. Fluid flow is modified because of mechanical forces arising in view of magnetic field on these currents. MHD compressor operation, blood pump machines, design of heat exchangers, flow meters, power generators, radar systems etc are based upon MHD principles. Such principles in bioengineering and medical sciences have been utilized for targeted drugs transport, bleeding reduction during surgeries, magnetic devices development for cell separation, magnetic tracers development, hyperthermia etc. Magneto therapy largely involve MHD non-Newtonian materials. In particular, the MHD peristaltic flows have relevance for problems about urinary tract, cells and tissues behavior modification and cure of gastrointestinal motility related disorders. Having all such in mind, some advancements have been made for peristaltic flows of MHD fluids in a channel (see [3–15]). It should be noted that much alteration in the past has been focused to such flows using constant applied magnetic field. Distinct from the previous studies are in present attempt will deal with the peristalsis through non-uniform applied magnetic field in the radial direction. We believe that such consideration in the peristaltic flows of physiological fluids is more realistic.

Impact of heat transfer in peristaltic transport of fluid is quite significant in food processing, oxygenation, hemodialysis, tissues conduction, heat convection for blood flow from the pores of tissues and radiation between environment and its surface. Mass transfer is useful in the aforementioned processes. Especially mass transfer cannot be under estimated when nutrients diffuse out from the blood to neighboring tissues. Further mass transfer involvement is quitee prevalent in distillation, chemical impurities diffusion, membrane separation and combustion process. It should be noted that relationships between fluxes and driving potentials occur when both heat and mass transfer act simultaneously. Here temperature gradient generates energy flux. However mass flux and composition gradients are due to temperature gradient (which is called Soret effect). Thermal diffusion (or Dufour effect) is the energy flux induced by concentration gradient. Although sizeable information exists about peristaltic flows in presence of heat and mass transfer but Soret and Dufour effects are less emphasized (see [16–25]).

Previous literature on the topic witnesses that peristaltic flows of fluids in a curved geometrical configurations have been scarcely examined. To our knowledge there are only few attempts [26–34] which address this aspect. Also there is not any attempt available which investigates the effect of radial magnetic field on the peristaltic flow of Jeffrey liquid in a curved complaint wall channel. The objective here is to address this problem. Thus relevant equations are modeled. These equations are then reduced subject to lubrication approach. The resulting problems for heat and mass transfer in a curved channel with convective conditions are also considered. Function formulation are adopted. Stream results for stream function, temperature, concentration and heat transfer coefficient are obtained and discussed. Streamlines are plotted and studied.

## Problem development

**Fig 1:**
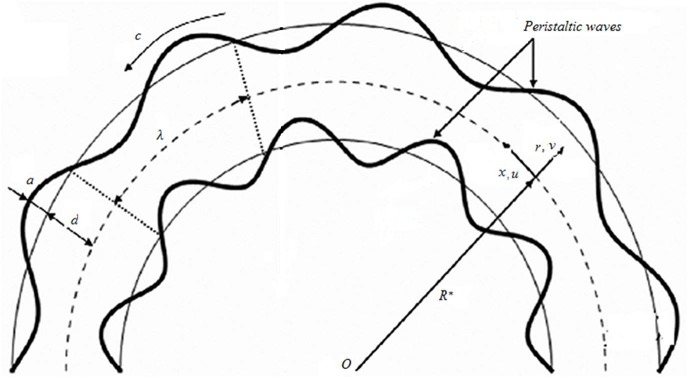
Schematic picture.

Consider an incompressible Jeffrey liquid in curved channel of thickness 2*d* and mean radius R*. The wave is propagating along the walls of channel with velocity *c*. Let *u*(*r*, *x*, *t*) and *v*(*r*, *x*, *t*) represent the components of velocity in the axial *x* and radial *r* directions respectively (see refs. [28] and [29]). The peristaltic wave shape is represented by

$$\begin{array}{c} r=\pm \eta \left(x,t\right)=\pm [d+a\sin\frac{2\pi }{\lambda }\left(x-ct\right)], \end{array}$$ (1)

and the physical geometry of the problem is given by Fig 1

where *a* is the amplitude of wave, *λ* the wavelength and *t* the time. The displacements of the upper and lower walls are represented by ±*η* respectively. Further in radial direction we applied a magnetic field **B** by which the fluid is electrically conducting. The magnetic field in radial direction is defined by

$$\begin{array}{c} B=\frac{B_{0}}{r+R^{*}}e_{r}, \end{array}$$ (2)

where *B*_0_ shows the magnetic field strength and *e*_*r*_ is unit vector in the radial direction. Utilization of Ohm’s law gives the following expression

$$\begin{array}{c} J\times B=\frac{-\sigma B_{0}^{2}u}{{\left(r+R^{*}\right)}^{2}}e_{x}, \end{array}$$ (3)

in which **J** represents the current density, *σ* the electrical conductivity, *u* the velocity component in axial direction and *e*_*x*_ corresponds to unit vector in axial direction.

The constitutive equations for Jeffrey model are given by [9]:

$$\begin{array}{ccc} \tau & = & -pI+S,\\ S & = & \frac{\mu }{1+\beta }\left[1+\lambda_{3}\frac{d}{dt}\right]A_{1}, \end{array}$$ (4)

$$\begin{array}{ccc} A_{1} & = & \nabla V+{\left(\nabla V\right)}^{transpose} \end{array}$$ (5)

and in component form, **S** can be written as follows:

$$\begin{array}{ccc} S_{rr} & = & \frac{2\mu }{1+\beta }\left[1+\lambda_{3}\left(\frac{\partial }{\partial t}+v\frac{\partial }{\partial r}+\frac{R^{*}u}{r+R^{*}}\frac{\partial }{\partial x}\right)\right]\frac{\partial v}{\partial r},\\ S_{xr} & = & S_{rx}=\frac{2\mu }{1+\beta }\left[1+\lambda_{3}\left(\frac{\partial }{\partial t}+v\frac{\partial }{\partial r}+\frac{R^{*}u}{r+R^{*}}\frac{\partial }{\partial x}\right)\right]\left(\frac{\partial u}{\partial r}+\frac{R^{*}}{r+R^{*}}\frac{\partial v}{\partial x}-\frac{u}{r+R^{*}}\right),\\ S_{xx} & = & \frac{2\mu }{1+\beta }\left[1+\lambda_{3}\left(\frac{\partial }{\partial t}+v\frac{\partial }{\partial r}+\frac{R^{*}u}{r+R^{*}}\frac{\partial }{\partial x}\right)\right]\left(\frac{R^{*}}{r+R^{*}}\frac{\partial u}{\partial x}+\frac{v}{r+R^{*}}\right), \end{array}$$ (6)

where *τ* is the Cauchy stress tensor, *p* the pressure, **I** the identity tensor, **S** the extra stress tensor, **A**_1_ the first Rivlin Ericksen tensor, *μ* the fluid dynamic viscosity, *β* ratio of the relaxation to retardation times and *λ*_3_ the retardation time.

The governing equations in absence of body forces are given by

Equation of Continuity:

$$\begin{array}{c} \frac{\partial v}{\partial r}+\frac{R^{*}}{r+R^{*}}\frac{\partial u}{\partial x}+\frac{v}{r+R^{*}}=0, \end{array}$$ (7)

Component of momentum equation in radial direction:

$$\begin{array}{c} \rho \left[\frac{\partial v}{\partial t}+v\frac{\partial v}{\partial r}+\frac{R^{*}u}{r+R^{*}}\frac{\partial v}{\partial x}-\frac{u^{2}}{r+R^{*}}\right]=-\frac{\partial p}{\partial r}+\mu \frac{\partial }{\partial r}\left(S_{rr}\right)+\frac{\mu R^{*}}{r+R^{*}}\frac{\partial }{\partial x}\left(S_{rx}\right)-\frac{\mu }{r+R^{*}}\left(S_{xx}\right). \end{array}$$ (8)

Component of momentum equation in axial direction:

$$\begin{array}{ccc} \rho [\frac{\partial u}{\partial t}+v\frac{\partial u}{\partial r}+\frac{R^{*}u}{r+R^{*}}\frac{\partial u}{\partial x}+\frac{uv}{r+R^{*}}] & = & -\frac{R^{*}}{r+R^{*}}\frac{\partial p}{\partial x}+\mu \frac{\partial }{\partial r}\left(S_{rx}\right)+\frac{\mu R^{*}}{r+R^{*}}\frac{\partial }{\partial x}\left(S_{xx}\right)\\ & & -\frac{\sigma B_{0}^{2}u}{{\left(r+R^{*}\right)}^{2}}. \end{array}$$ (9)

Energy equation comprising viscous dissipation and Dufour effects:

$$\begin{array}{ccc} \rho c_{p}\left[\frac{\partial }{\partial t}+v\frac{\partial }{\partial r}+\frac{R^{*}u}{r+R^{*}}\frac{\partial }{\partial x}\right]T & = & \kappa_{t}\left[\frac{\partial^{2}T}{\partial r^{2}}+\frac{1}{r+R^{*}}\frac{\partial T}{\partial r}+\left(\frac{R^{*}}{r+R^{*}}\right)\frac{\partial^{2}T}{\partial x^{2}}\right]\\ & & +\frac{Dk_{T}}{C_{S}}\left[\frac{\partial^{2}C}{\partial r^{2}}+\frac{1}{r+R^{*}}\frac{\partial C}{\partial r}+\left(\frac{R^{*}}{r+R^{*}}\right)\frac{\partial^{2}C}{\partial x^{2}}\right]+tr\left(\text{LS}\right), \end{array}$$ (10)

Concentration equation with Soret effect is

$$\begin{array}{ccc} {}[\frac{\partial }{\partial t}+v\frac{\partial }{\partial r}+\frac{R^{*}u}{r+R^{*}}\frac{\partial }{\partial x}]C & = & D(\frac{\partial^{2}C}{\partial r^{2}}+\frac{1}{r+R^{*}}\frac{\partial C}{\partial r}+\left(\frac{R^{*}}{r+R^{*}}\right)\frac{\partial^{2}C}{\partial x^{2}})\\ & & +\frac{Dk_{T}}{T_{m}}\left(\frac{\partial^{2}T}{\partial r^{2}}+\frac{1}{r+R^{*}}\frac{\partial T}{\partial r}+\left(\frac{R^{*}}{r+R^{*}}\right)\frac{\partial^{2}T}{\partial x^{2}}\right). \end{array}$$ (11)

In above equations *ρ* the density of fluid, *ν* the kinematic viscosity, *T* the fluid temperature, *C* the concentration of fluid, *T*_0_ and *C*_0_ the temperature and concentration at the lower and upper walls respectively, *c*_*p*_ the specific heat at constant pressure, *C*_*S*_ the concentration susceptibility, *D* the mass diffusivity coefficient, *k*_*T*_ the thermal diffusion ratio and *κ*_*t*_ the thermal conductivity. Here **L** = ∇**V** and *T*_*m*_ the mean fluid temperature.

The convective boundary conditions for the exchange of heat and concentration, no slip condition and compliant nature of the walls are described through the expressions

$$\begin{array}{ccc} \kappa_{t}\frac{\partial T}{\partial r} & = & -h_{1}\left(T-T_{0}\right),\;\text{at}\;r=\eta ,\\ \kappa_{t}\frac{\partial T}{\partial r} & = & -h_{1}\left(T_{0}-T\right),\;\text{at}\;r=-\eta , \end{array}$$ (12)

$$\begin{array}{c} u=0,\;\text{at}\;r=\pm \eta , \end{array}$$ (13)

$$\begin{array}{ccc} D\frac{\partial C}{\partial r} & = & -h_{2}\left(C-C_{0}\right),\;\text{at}\;r=\eta ,\\ D\frac{\partial C}{\partial r} & = & -h_{2}\left(C_{0}-C\right),\;\text{at}\;r=-\eta , \end{array}$$ (14)

$$\begin{array}{ccc} \frac{R^{*}}{r+R^{*}}\left[-\tau \frac{\partial^{3}}{\partial x^{3}}+m_{1}^{*}\frac{\partial^{3}}{\partial x\partial t^{2}}+d^{\prime }\frac{\partial^{2}}{\partial t\partial x}\right]\eta =-\rho \left[\frac{\partial u}{\partial t}+v\frac{\partial u}{\partial r}+\frac{R^{*}u}{r+R^{*}}\frac{\partial u}{\partial x}+\frac{uv}{r+R^{*}}\right] & & \\ +{\left(\frac{1}{r+R^{*}}\right)}^{2}\frac{\partial }{\partial r}\left({\left(r+R^{*}\right)}^{2}S_{rx}\right)+\frac{R^{*}}{r+R^{*}}\frac{\partial }{\partial x}\left(S_{xx}\right) & & \\ -\frac{\sigma B_{0}^{2}u}{{\left(r+R^{*}\right)}^{2}},\;\text{at}\;r=\pm \eta . & \end{array}$$ (15)

In above expressions *h*_1_ and *h*_2_ are the heat and mass transfer coefficients at the upper and lower walls of the channel respectively, *τ* the elastic tension in the membrane, $m_{1}^{*}$ the mass per unit area and *d*′ the coefficient of viscous damping.

On setting velocity in terms of stream function in polar cordinates

$$\begin{array}{c} u=-\frac{\partial \psi }{\partial r},\;v=\frac{R^{*}}{r+R^{*}}\frac{\partial \psi }{\partial x} \end{array}$$ (16)

and using the non-dimensional variables

$$\begin{array}{ccc} \psi^{*} & = & \frac{\psi }{cd},\;x^{*}=\frac{x}{\lambda },\;r^{*}=\frac{r}{d},t^{*}=\frac{ct}{\lambda },\;u^{*}=\frac{u}{c},\\ v^{*} & = & \frac{v}{c}\eta^{*}=\frac{\eta }{d},\;\theta =\frac{T-T_{0}}{T_{0}},\phi =\frac{C-C_{0}}{C_{0}},\;p^{*}=\frac{d^{2}p}{\mu c\lambda },\\ \;k & = & \frac{R^{*}}{d},\;S_{xr}=S_{rx}=\frac{\mu c}{d}\;S_{xr}^{*},\;S_{xx}=\frac{\mu c}{d}S_{xx}^{*},\;S_{rr}=\frac{\mu c}{d}S_{rr}. \end{array}$$ (17)

The long wavelength and low Reynolds number assumptions are commonly used in the analysis of peristalsis flow [35–38]. Using this approach Eqs (5)–(10) becomes

$$\begin{array}{c} \frac{\partial p}{\partial r}=0, \end{array}$$ (18)

$$\begin{array}{c} \frac{-k}{r+k}\frac{\partial p}{\partial x}+\frac{1}{{\left(r+k\right)}^{2}}\frac{\partial }{\partial r}\left[{\left(r+k\right)}^{2}S_{xr}\right]+H^{2}\frac{\psi_{r}}{{\left(r+k\right)}^{2}}=0, \end{array}$$ (19)

$$\begin{array}{c} \frac{\partial^{2}\theta }{\partial r^{2}}+\frac{1}{r+k}\frac{\partial \theta }{\partial r}+\mathrm{Pr}Du\left(\frac{\partial^{2}\phi }{\partial r^{2}}+\frac{1}{r+k}\frac{\partial \phi }{\partial r}\right)+Br\left(\frac{1}{r+k}\frac{\partial \psi }{\partial r}-\frac{\partial^{2}\psi }{\partial r^{2}}\right)S_{xr}=0, \end{array}$$ (20)

$$\begin{array}{c} \frac{\partial^{2}\phi }{\partial r^{2}}+\frac{1}{r+k}\frac{\partial \phi }{\partial r}+ScSr\left(\frac{\partial^{2}\theta }{\partial r^{2}}+\frac{1}{r+k}\frac{\partial \theta }{\partial r}\right)=0, \end{array}$$ (21)

with the dimensionless conditions

$$\begin{array}{ccc} \eta & = & 1+\epsilon \sin2\pi (x-t), \end{array}$$ (22)

$$\begin{array}{ccc} \frac{\partial \theta }{\partial r}+Bi_{1}\theta & = & 0\;\text{at}\;r=\eta ,\\ \frac{\partial \theta }{\partial r}-Bi_{1}\theta & = & 0\;\text{at}\;r=-\eta , \end{array}$$ (23)

$$\begin{array}{ccc} \psi_{r} & = & 0\;\text{at}\;r=\pm \eta , \end{array}$$ (24)

$$\begin{array}{ccc} \frac{\partial \phi }{\partial r}+Bi_{2}\phi & = & 0\;\text{at}\;r=\eta ,\\ \frac{\partial \phi }{\partial r}-Bi_{2}\phi & = & 0\;\text{at}\;r=-\eta , \end{array}$$ (25)

$$\begin{array}{ccc} \frac{k}{r+k}\left[E_{1}\frac{\partial^{3}}{\partial x^{3}}+E_{2}\frac{\partial^{3}}{\partial x\partial t^{2}}+E_{3}\frac{\partial^{2}}{\partial x\partial t}\right]\eta & = & \frac{1}{{\left(r+k\right)}^{2}}\frac{\partial }{\partial r}\left[{\left(r+k\right)}^{2}S_{xr}\right]\\ +\frac{H^{2}}{{\left(r+k\right)}^{2}}\psi_{r}\;\text{at}\;r & = & \pm \eta . \end{array}$$ (26)

In above equations, *δ* corresponds to the dimensionless wave number, *Re* the Reynolds number, Pr the Prandtl number, *H* the Hartmann number, *E*_*i*_(*i* = 1 − 3) the non-dimensional elasticity parameters, *E* the Eckert number, *Br* the Brinkman number, *Sr* the Soret number, *Du* the Dufour number, *ϵ* the amplitude ratio parameter and $Bi_{\hat{ı}}\left(\hat{ı}=1,2\right)$ the heat and mass transfer Biot numbers respectively. The values of these parameters can be defined as follows:

$$\begin{array}{ccc} \delta & = & \frac{d}{\lambda },\;\text{Re}=\frac{cd}{\nu },\;\mathrm{Pr}=\frac{\mu c_{p}}{\kappa_{t}},\;\epsilon =\frac{a}{d},\;E=\frac{c^{2}}{T_{0}c_{p}},\\ E_{1} & = & -\frac{\tau d^{3}}{\lambda^{3}\mu c},\;E_{2}=\frac{m_{1}^{*}cd^{3}}{\lambda^{3}\mu },\;E_{3}=\frac{d^{3}d^{\prime }}{\mu \lambda^{2}},Br=EPr,\;Sc=\frac{\mu }{\rho D},\\ Bi_{1} & = & \frac{h_{1}d}{\kappa_{t}},\;Bi_{2}=\frac{h_{2}d}{D},\;H^{2}=\frac{\sigma B_{0}^{2}}{\mu },\;Sr=\frac{\rho Dk_{T}T_{0}}{\mu T_{m}C_{0}},\;Du=\frac{Dk_{T}C_{0}}{\mu C_{s}C_{p}T_{0}}. \end{array}$$ (27)

Through Eqs (17) and (18) we have

$$\begin{array}{c} \frac{\partial }{\partial r}\left[\frac{1}{{\left(r+k\right)}^{2}}\frac{\partial }{\partial r}\left[{\left(r+k\right)}^{2}S_{xr}\right]+H^{2}\frac{\psi_{r}}{{\left(r+k\right)}^{2}}\right]=0. \end{array}$$ (28)

where non-dimensional tensor is

$$\begin{array}{c} S_{xr}=\frac{2}{1+\beta }\left[-\psi_{rr}+\frac{\psi_{r}}{r+k}\right]. \end{array}$$

### Method of solution

The closed form solutions of Eqs (20), (21) and (28) are

$$\begin{array}{c} \psi ={\left(k+r\right)}^{1+\sqrt{1+H^{2}\left(1+\beta \right)}}D_{1}+{\left(k+r\right)}^{1-\sqrt{1+H^{2}\left(1+\beta \right)}}D_{2}+krD_{3}+\frac{1}{2}r^{2}D_{3}+D_{4}, \end{array}$$ (29)

$$\begin{array}{c} \theta =C_{1}^{2}C_{3}{\left(k+r\right)}^{2\sqrt{1+H^{2}+\beta H^{2}}}+C_{2}^{2}C_{4}{\left(k+r\right)}^{-2\sqrt{1+H^{2}+\beta H^{2}}}A_{2}+A_{1}Log\left[k+r\right]-C_{1}C_{2}C_{5}Log{\left[k+r\right]}^{2}, \end{array}$$ (30)

$$\begin{array}{c} \phi =B_{1}\theta \left[r\right]+B_{2}Log\left[k+r\right]+B_{3} \end{array}$$ (31)

and the heat transfer coefficient is defined by

$$\begin{array}{c} Z=\eta_{x}\theta_{r}\left(\eta \right), \end{array}$$

where *A*_*i*_(*i* = 1 − 2), *B*_*j*_(*j* = 1 − 3), *D*_*l*_(*l* = 1 − 4), *C*_*m*_(*m* = 1 − 5) and *Z* are mentioned in appendix.

## Results and discussion

In this section the results of velocity, temperature, concentration, heat transfer coefficient and streamlines are discussed physically.

### 0.1 Velocity profile

**Fig 2:**
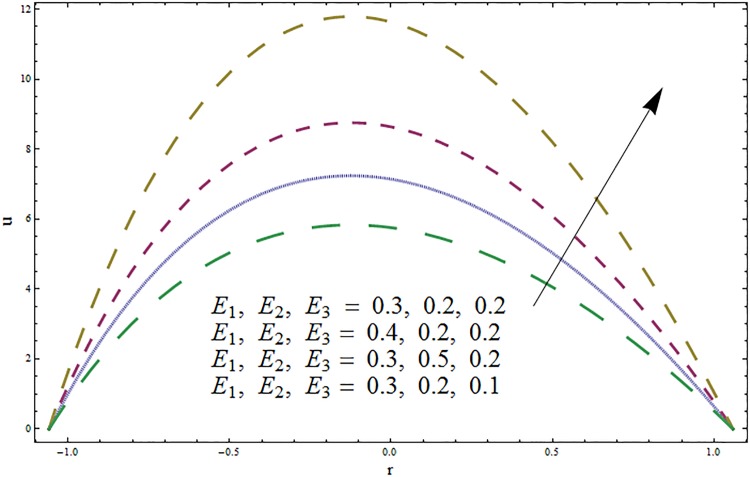
Variation in velocity *u* for wall parameters *E*_1_, *E*_2_, *E*_3_ when *ϵ* = 0.1, *t* = 0.2, *x* = 0.3, *k* = 3, *β* = 0.4 and *H* = 0.5.

**Fig 3:**
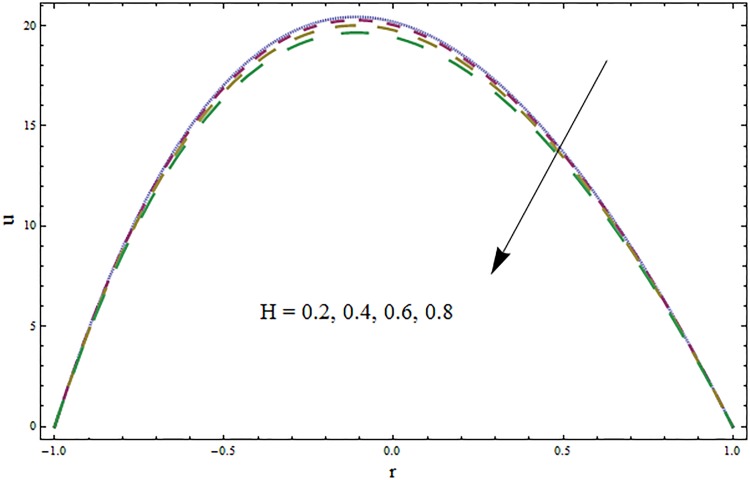
Variation in velocity *u* for Hartmann number *H* when *ϵ* = 0.1, *t* = 0.2, *x* = 0.2, *k* = 3, *E*_1_ = 0.9, *E*_2_ = 0.3, *E*_3_ = 0.1 and *β* = 0.4.

**Fig 4:**
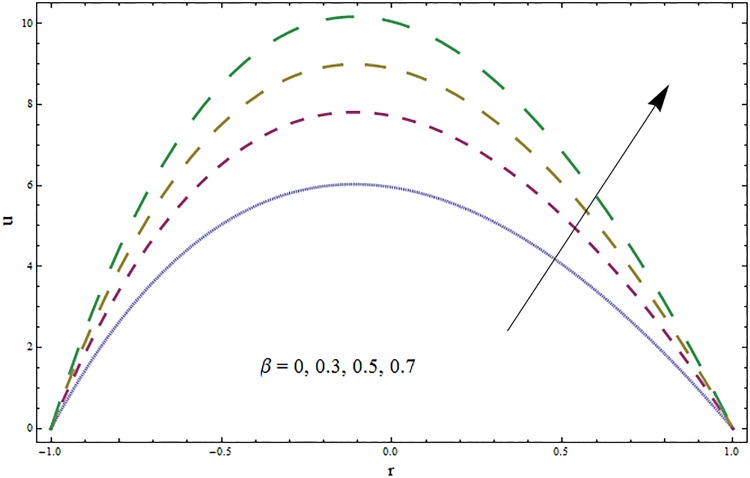
Variation in velocity *u* for ratio of relaxation and retardation times *β* when *ϵ* = 0.1, *t* = 0.2, *x* = 0.2, *k* = 3, *E*_1_ = 0.3, *E*_2_ = 0.2, *E*_3_ = 0.1 and *H* = 0.5.

**Fig 5:**
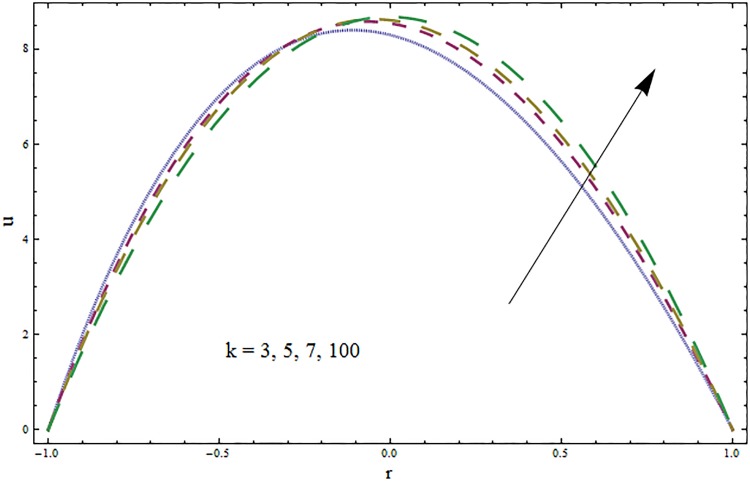
Variation in velocity *u* for curvature parameter *k* when *ϵ* = 0.1, *t* = 0.2, *x* = 0.2, *k* = 3, *E*_1_ = 0.3, *E*_2_ = 0.2, *E*_3_ = 0.1 and *H* = 0.5.

The impacts of elasticity parameters *E*_1_, *E*_2_ and *E*_3_ on the velocity distribution is presented in Fig 2. The obtained results illustrate the increase in velocity via larger wall parameters *E*_1_ and *E*_2_. The velocity decays when parameter *E*_3_ is decreased. Because of elasticity of walls there is less resistance to flow and thus velocity increases. The variations of Hartmann number *H* on velocity profile are depicted in Fig 3. Velocity profile decreases due to an applied magnetic field. This magnetic field creates a resistive force similar to drag force that acts in opposite direction of fluid motion. The results for the ratio of relaxation and retardation times *β* are presented in Fig 4. The graph indicates the increase in velocity when the ratio of relaxation and retardation times *β* enhances. It is possible only when there is increase in relaxation time and decrease in retardation time. Fig 5 indicates an increase in velocity for larger curvature parameter. In fact through increase in curvature parameter the velocity is tilted towards lower wall.

### 0.2 Temperature

**Fig 6:**
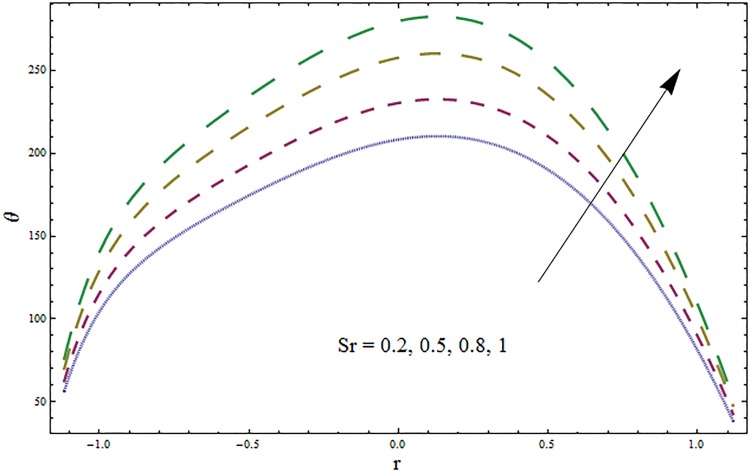
Variation in temperature *θ* for Soret number *Sr* when *ϵ* = 0.2, *t* = 0.1, *x* = 0.2, *k* = 2, *E*_1_ = 0.3, *E*_2_ = 0.2, *E*_3_ = 0.1, *Br* = 2, *β* = 0.5, *Bi*_1_ = 10, *Bi*_2_ = 0.5, *H* = 0.8, *Du* = 0.5, *Pr* = 2 and *Sc* = 0.3.

**Fig 7:**
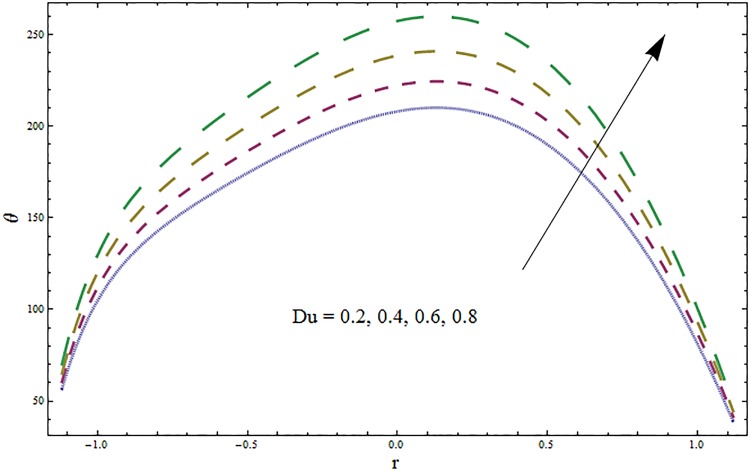
Variation in temperature *θ* for Dufour number *Du* when *ϵ* = 0.2, *t* = 0.1, *x* = 0.2, *k* = 2, *E*_1_ = 0.3, *E*_2_ = 0.2, *E*_3_ = 0.1, *Br* = 2, *β* = 0.5, *Bi*_1_ = 10, *Bi*_2_ = 0.5, *Sr* = 0.5, *Pr* = 2, *Sc* = 0.3 and *H* = 0.8.

**Fig 8:**
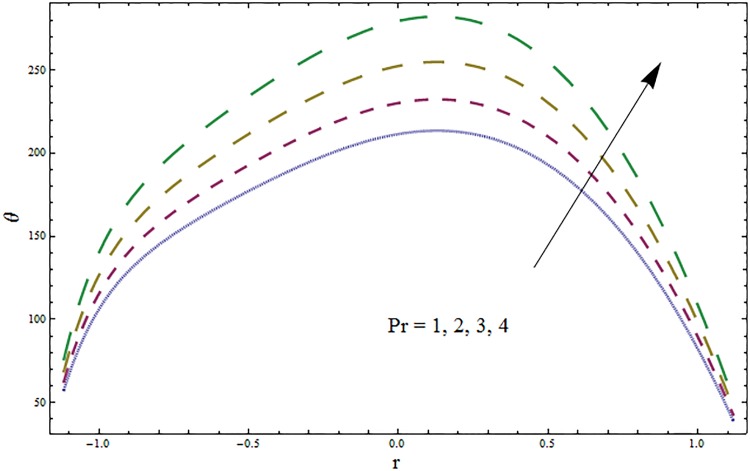
Variation in temperature *θ* for Prandtl number Pr when *ϵ* = 0.2, *t* = 0.1, *x* = 0.2, *k* = 2, *E*_1_ = 0.3, *E*_2_ = 0.2, *E*_3_ = 0.1, *Br* = 2, *β* = 0.5, *Bi*_1_ = 10, *Bi*_2_ = 0.5, *H* = 0.8, *Du* = 0.5, *Sr* = 0.5 and *Sc* = 0.3.

**Fig 9:**
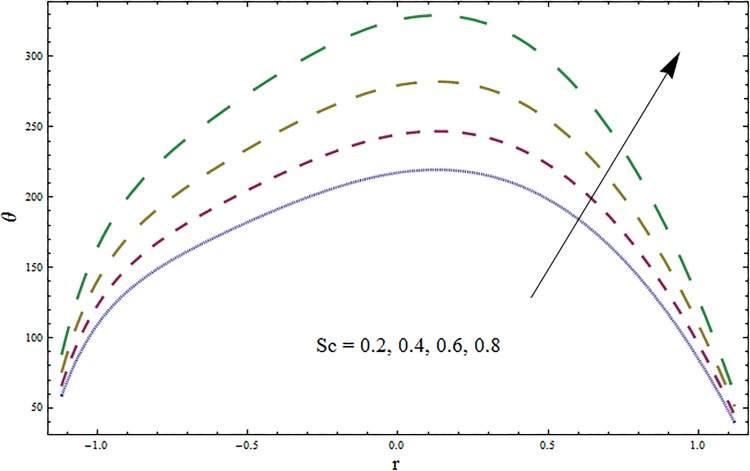
Variation in temperature *θ* for Schmidt number *Sc* when *ϵ* = 0.2, *t* = 0.1, *x* = 0.2, *k* = 2, *E*_1_ = 0.3, *E*_2_ = 0.2, *E*_3_ = 0.1, *Br* = 2, *β* = 0.5, *Bi*_1_ = 10, *Bi*_2_ = 0.5, *H* = 0.8, *Du* = 0.5, *Sr* = 0.5 and Pr = 2.

**Fig 10:**
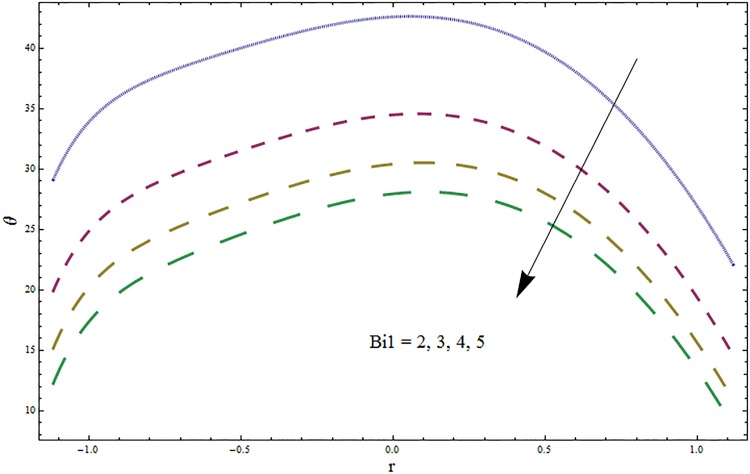
Variation in temperature *θ* for heat transfer Biot number *Bi*_1_ when *ϵ* = 0.2, *t* = 0.1, *x* = 0.2, *k* = 2, *E*_1_ = 0.3, *E*_2_ = 0.2, *E*_3_ = 0.1, *Br* = 2, *β* = 0.5, *Sc* = 0.3, *Bi*_2_ = 0.5, *H* = 0.8, *Du* = 0.5, *Sr* = 0.5 and Pr = 2.

**Fig 11:**
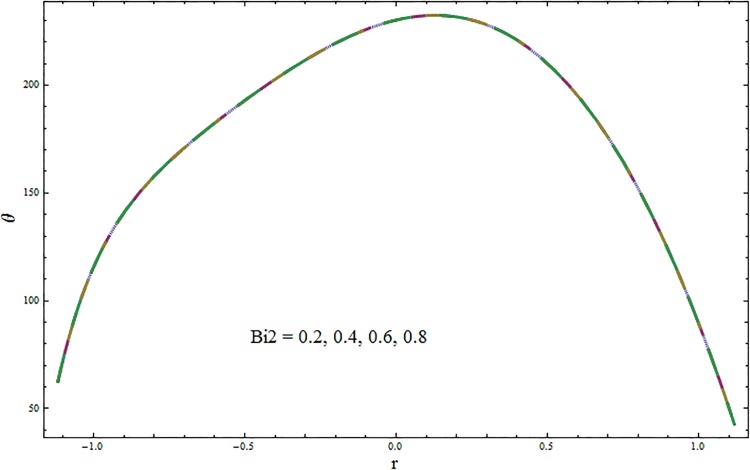
Variation in temperature *θ* for mass transfer Biot number *Bi*_2_ when *ϵ* = 0.2, *t* = 0.1, *x* = 0.2, *k* = 2, *E*_1_ = 0.3, *E*_2_ = 0.2, *E*_3_ = 0.1, *Br* = 2, *β* = 0.5, *Bi*_1_ = 10, *Sc* = 0.3, *H* = 0.8, *Du* = 0.5, *Sr* = 0.5 and Pr = 2.

**Fig 12:**
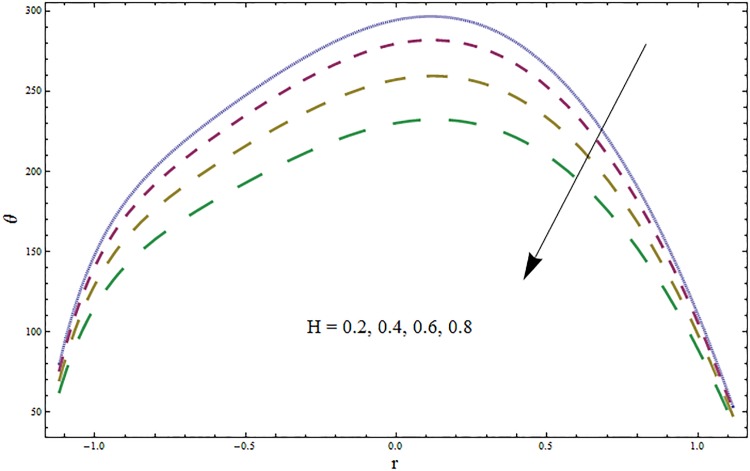
Variation in temperature *θ* for Hartmann number *H* when *ϵ* = 0.2, *t* = 0.1, *x* = 0.2, *k* = 2, *E*_1_ = 0.3, *E*_2_ = 0.2, *E*_3_ = 0.1, *Br* = 2, *β* = 0.5, *Bi*_1_ = 10, *Sc* = 0.3, *Bi*_2_ = 0.5, *Du* = 0.5, *Sr* = 0.5 and Pr = 2.

**Fig 13:**
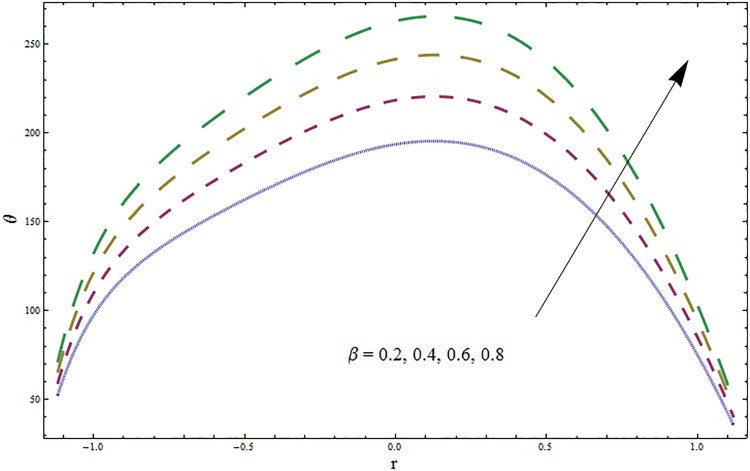
Variation in temperature *θ* for ratio of relaxation and retardation times parameter *β* when *ϵ* = 0.2, *t* = 0.1, *x* = 0.2, *k* = 2, *E*_1_ = 0.3, *E*_2_ = 0.2, *E*_3_ = 0.1, *Br* = 2, *H* = 0.8, *Bi*_1_ = 10, *Sc* = 0.3, *Bi*_2_ = 0.5, *Du* = 0.5, *Sr* = 0.5 and Pr = 2.

**Fig 14:**
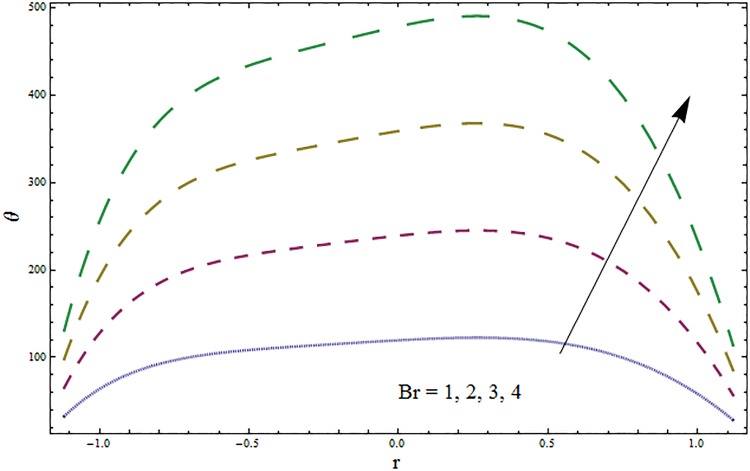
Variation in temperature *θ* for Brinkman number *Br* when *ϵ* = 0.2, *t* = 0.1, *x* = 0.2, *k* = 2, *E*_1_ = 0.3, *E*_2_ = 0.2, *E*_3_ = 0.1, *β* = 0.5, *H* = 0.8, *Bi*_1_ = 10, *Sc* = 0.3, *Bi*_2_ = 0.5, *Du* = 0.5, *Sr* = 0.5 and Pr = 2.

**Fig 15:**
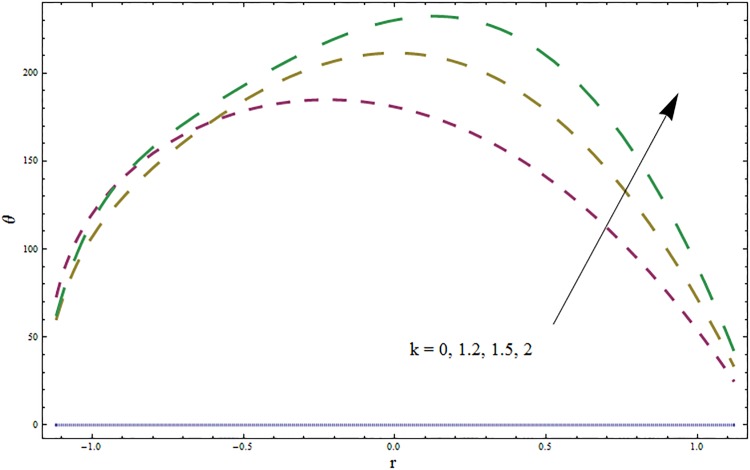
Variation in temperature *θ* for curvature parameter *k* when *ϵ* = 0.2, *t* = 0.1, *x* = 0.2, *Br* = 2, *E*_1_ = 0.3, *E*_2_ = 0.2, *E*_3_ = 0.1, *β* = 0.5, *H* = 0.8, *Bi*_1_ = 10, *Sc* = 0.3, *Bi*_2_ = 0.5, *Du* = 0.5, *Sr* = 0.5 and Pr = 2.

**Fig 16:**
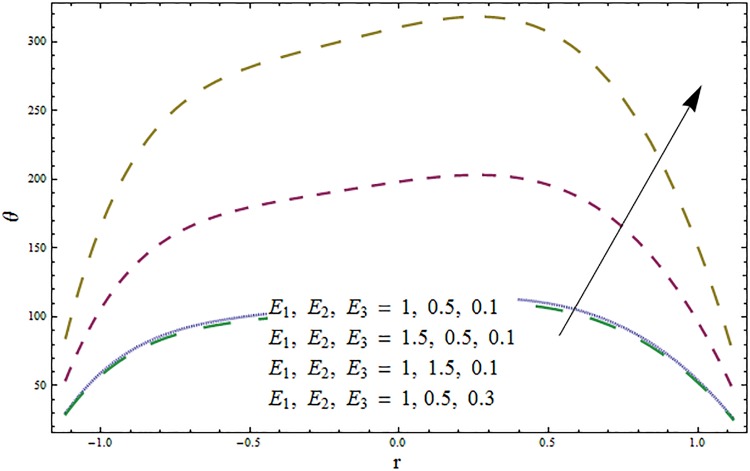
Variation in temperature *θ* for wall parameters *E*_1_, *E*_2_, *E*_3_ when *ϵ* = 0.2, *t* = 0.1, *x* = 0.2, *k* = 5, *β* = 0.5, *H* = 0.8, *Bi*_1_ = 10, *Sc* = 0.3, *Bi*_2_ = 0.5, *Du* = 0.5, *Sr* = 0.5, Pr = 2 and *H* = 0.5.

Figs 6–16 are developed for the physical interpretation of embedded parameters on temperature distribution *θ*. From Figs 6 and 7 it is observed that temperature rises inside the channel for Dufour *Du* and Soret *Sr* effects. As increase in *Du* or *Sr* shows decrease in viscosity and so velocity increases. The fast moving fluid particles with larger molecular vibrations rises the temperature. The results plotted in Fig 8 perceive the increasing behavior of Prandtl number Pr due to increase in specific heat of fluid. Fig 9 shows an increase in temperature by increasing Schmidt number *Sc*. Clearly *Sc* causes reduction in viscosity. Decrease in temperature with increasing values of heat transfer Biot number *Bi*_1_ is seen through Fig 10. It is due to the fact that thermal conductivity of fluid reduces with an increase in *Bi*_1_ and so fluid temperature drops. On the other hand an increase in mass transfer Biot number *Bi*_2_ does not effect temperature of fluid (see Fig 11). The behavior of Hartmann number *H* is decreasing (see Fig 12). Since temperature and velocity are in direct relation so similar results are obtained. It is seen from Fig 13 that with an increase in ratio of relaxation and retardation times parameter *β* the temperature increases. Temperature profile is an increasing function of Brinkman number *Br* and curvature parameter *k* (see Figs 14 and 15). It is in view of the fact that Brinkman number involves the viscous dissipation effects that are responsible for the heat generation inside the channel. In Fig 16 the temperature increases for wall parameters which is similar to velocity profile. The results are found in good agreement with those obtained in limiting sense by Hina et al [28].

### 0.3 Heat transfer coefficient

**Fig 17:**
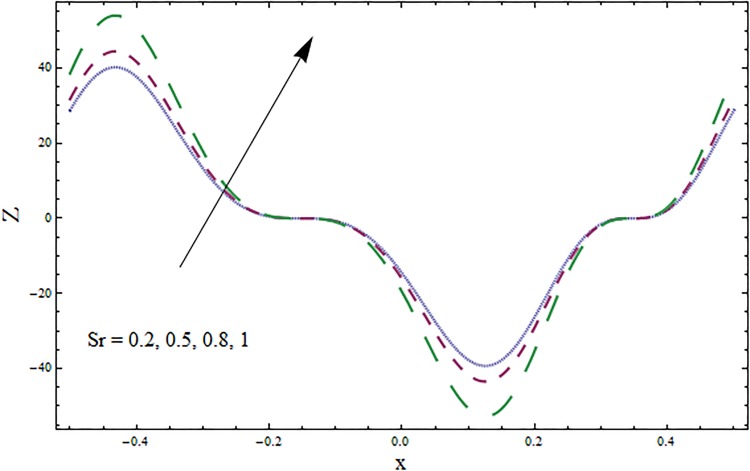
Variation in heat transfer coefficient *Z* for Soret number *Sr* when *ϵ* = 0.2, *t* = 0.1, *x* = 0.2, *k* = 5, *E*_1_ = 0.3, *E*_2_ = 0.2, *E*_3_ = 0.1, *β* = 0.5, *H* = 0.8, *Bi*_1_ = 10, *Sc* = 0.3, *Bi*_2_ = 0.5, *Du* = 0.5, *H* = 0.8 and Pr = 2.

**Fig 18:**
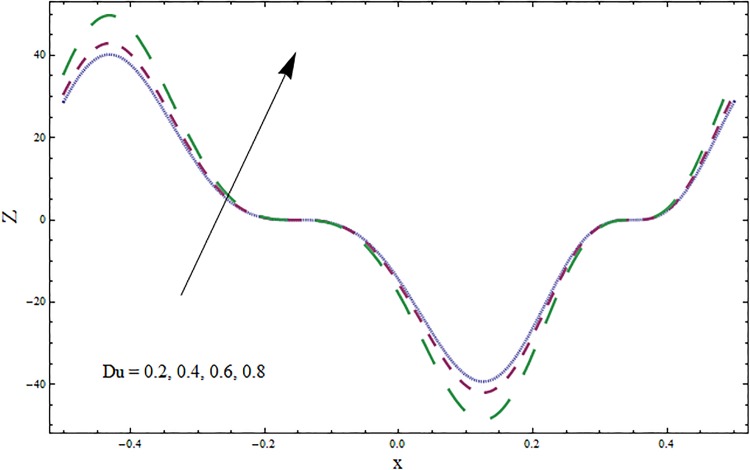
Variation in heat transfer coefficient *Z* for Dufour number *Du* when *ϵ* = 0.2, *t* = 0.1, *x* = 0.2, *k* = 2, *E*_1_ = 0.3, *E*_2_ = 0.2, *E*_3_ = 0.1, *β* = 0.5, *H* = 0.8, *Bi*_1_ = 10, *Sc* = 0.3, *Bi*_2_ = 0.5, *H* = 0.8, *Sr* = 0.5 and Pr = 2.

**Fig 19:**
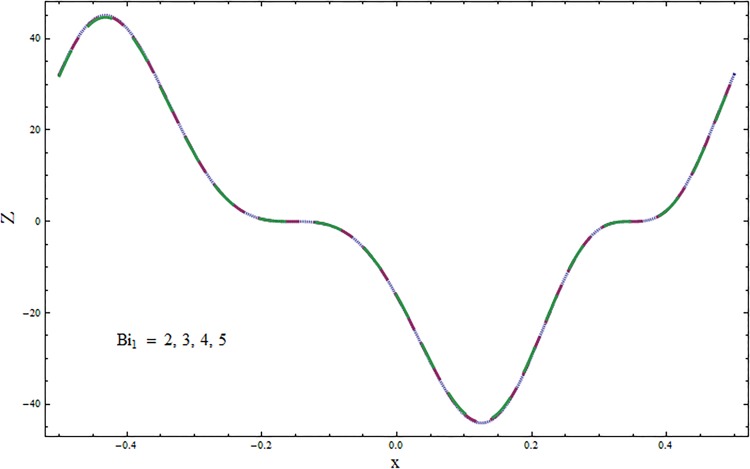
Variation in heat transfer coefficient *Z* for heat transfer Biot number *Bi*_1_ when *ϵ* = 0.2, *t* = 0.1, *x* = 0.2, *k* = 5, *E*_1_ = 0.3, *E*_2_ = 0.2, *E*_3_ = 0.1, *Br* = 0.1, *β* = 0.5, *Sc* = 0.3, *Bi*_2_ = 0.5, *H* = 0.8, *Du* = 0.5, *Sr* = 0.5 and Pr = 2.

**Fig 20:**
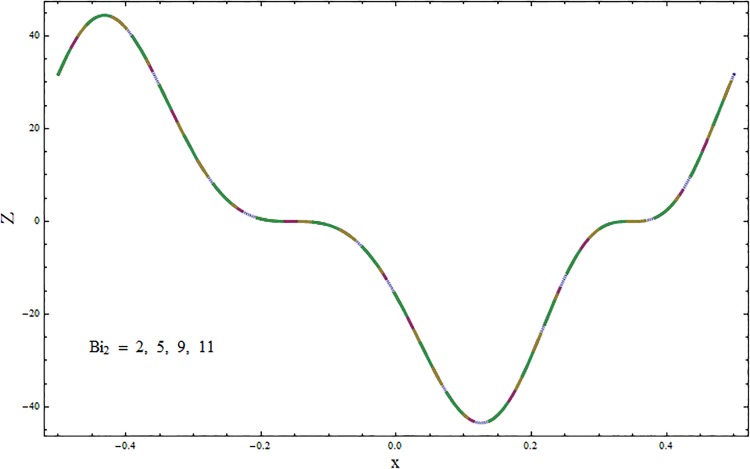
Variation in heat transfer coefficient *Z* for mass transfer Biot number *Bi*_2_ when *ϵ* = 0.2, *t* = 0.1, *x* = 0.2, *k* = 5, *E*_1_ = 0.3, *E*_2_ = 0.2, *E*_3_ = 0.1, *Br* = 0.1, *β* = 0.5, *Bi*_1_ = 10, *Sc* = 0.3, *H* = 0.8, *Du* = 0.5, *Sr* = 0.5 and Pr = 2.

**Fig 21:**
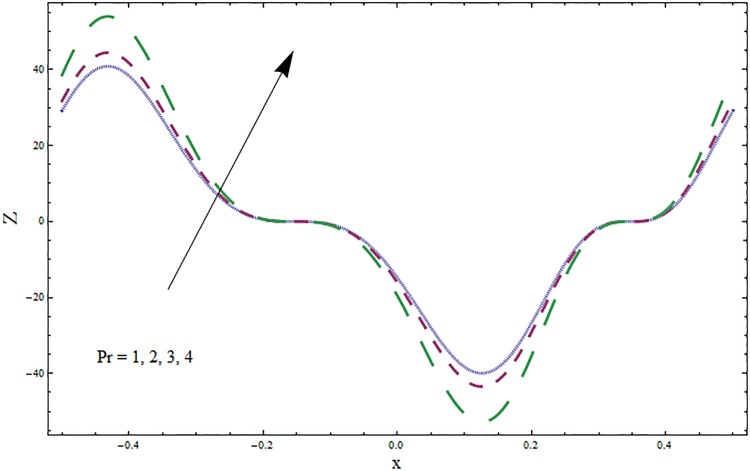
Variation in heat transfer coefficient *Z* for Prandtl number Pr when *ϵ* = 0.2, *t* = 0.1, *x* = 0.2, *k* = 5, *E*_1_ = 0.3, *E*_2_ = 0.2, *E*_3_ = 0.1, *Br* = 0.1, *β* = 0.5, *Bi*_1_ = 10, *Bi*_2_ = 0.5, *H* = 0.8, *Du* = 0.5, *Sr* = 0.5 and *Sc* = 0.3.

**Fig 22:**
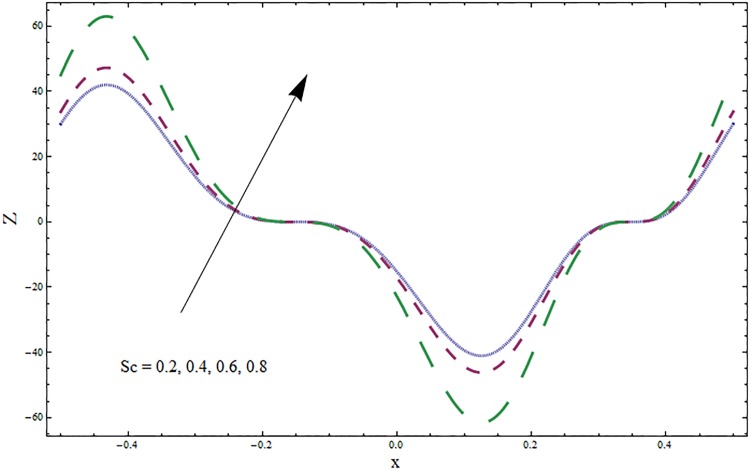
Variation in heat transfer coefficient *Z* for Schmidt number *Sc* when *ϵ* = 0.2, *t* = 0.1, *x* = 0.2, *k* = 5, *E*_1_ = 0.3, *E*_2_ = 0.2, *E*_3_ = 0.1, *Br* = 0.1, *β* = 0.5, *Bi*_1_ = 10, *Bi*_2_ = 0.5, *H* = 0.8, *Du* = 0.5, *Sr* = 0.5 and Pr = 2.

**Fig 23:**
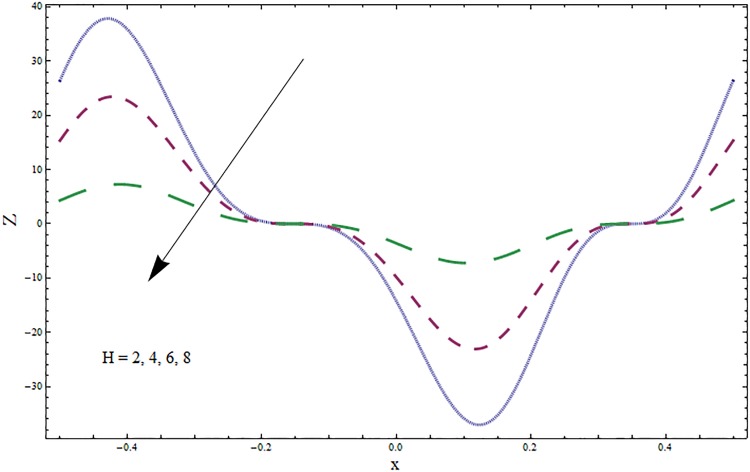
Variation in heat transfer coefficient *Z* for Hartmann number *H* when *ϵ* = 0.2, *t* = 0.1, *x* = 0.2, *k* = 5, *E*_1_ = 0.3, *E*_2_ = 0.2, *E*_3_ = 0.1, *Br* = 0.1, *β* = 0.5, *Bi*_1_ = 10, *Sc* = 0.3, *Bi*_2_ = 0.5, *Du* = 0.5, *Sr* = 0.5 and Pr = 2.

**Fig 24:**
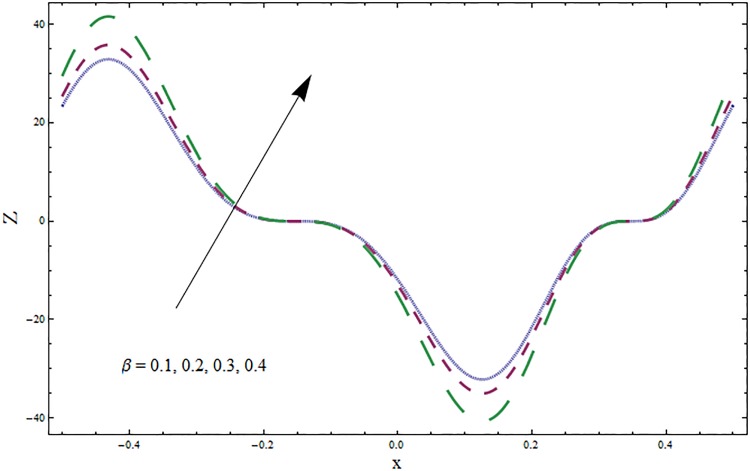
Variation in heat transfer coefficient *Z* for ratio of relaxation and retardation times parameter *β* when *ϵ* = 0.2, *t* = 0.1, *x* = 0.2, *k* = 5, *E*_1_ = 0.3, *E*_2_ = 0.2, *E*_3_ = 0.1, *Br* = 0.1, *H* = 0.8, *Bi*_1_ = 10, *Sc* = 0.3, *Bi*_2_ = 0.5, *Du* = 0.5, *Sr* = 0.5 and Pr = 2.

**Fig 25:**
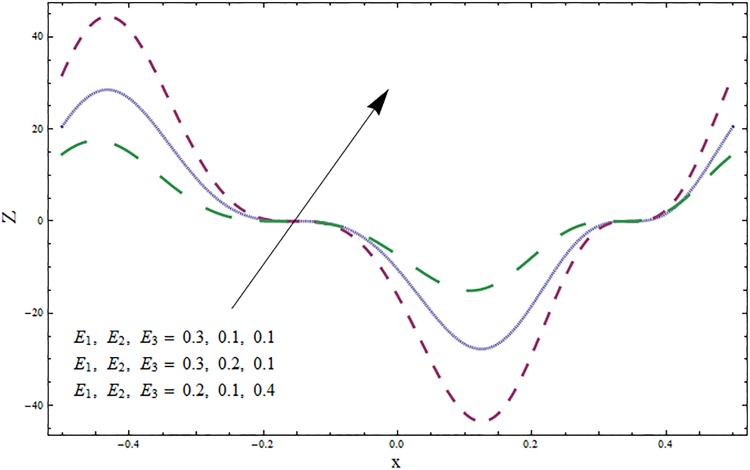
Variation in heat transfer coefficient *Z* for wall parameters *E*_1_, *E*_2_, *E*_3_ when *ϵ* = 0.2, *t* = 0.1, *x* = 0.2, *k* = 5, *Br* = 0.1, *β* = 0.5, *Bi*_1_ = 10, *Sc* = 0.3, *Bi*_2_ = 0.5, *Du* = 0.5, *Sr* = 0.5 and Pr = 2.

**Fig 26:**
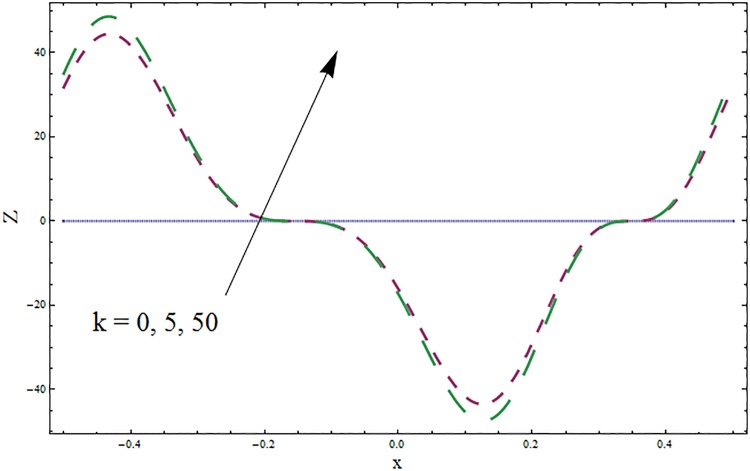
Variation in heat transfer coefficient *Z* for curvature parameter *k* when *ϵ* = 0.2, *t* = 0.1, *x* = 0.2, *β* = 0.5, *E*_1_ = 0.3, *E*_2_ = 0.2, *E*_3_ = 0.1, *Br* = 0.1, *H* = 0.8, *Bi*_1_ = 10, *Sc* = 0.3, *Bi*_2_ = 0.5, *Du* = 0.5, *Sr* = 0.5 and Pr = 2.

**Fig 27:**
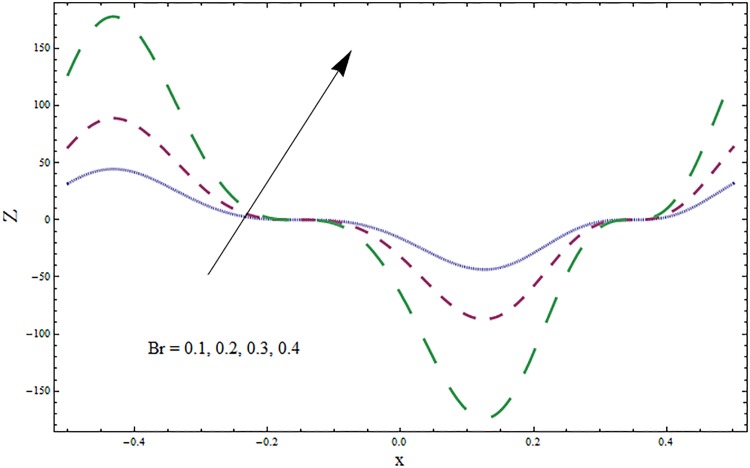
Variation in heat transfer coefficient *Z* for Brinkman number *Br* when *ϵ* = 0.2, *t* = 0.1, *x* = 0.2, *k* = 5, *E*_1_ = 0.3, *E*_2_ = 0.2, *E*_3_ = 0.1, *β* = 0.5, *H* = 0.8, *Bi*_1_ = 10, *Sc* = 0.3, *Bi*_2_ = 0.5, *Du* = 0.5, *Sr* = 0.5 and Pr = 2.

The graphs displayed in Figs 17–27 show oscillatory behavior of *Z* due to propagation of peristaltic waves. It is indicated from Figs 17 and 18 that for increasing Soret *Sr* and Dufour *Du* numbers the heat transfer increases inside the channel. The heat and mass transfer Biot numbers *Bi*_1_ and *Bi*_2_ give no net change on absolute value of heat transfer coefficient (see Figs 19 and 20). Figs 21 and 22 also show increasing behavior of heat transfer for larger Prandtl Pr and Schmidt *Sc* numbers respectively. The Hartmann number *H* reduces the magnitude of heat transfer coefficient *Z* near centerline of channel whereas it increases near the boundaries (see Fig 23). However *β* increases the heat transfer coefficient near centerline and it decreases along the boundary walls (see Fig 24). Fig 25 elucidates that magnitude of heat transfer coefficient enhances for wall parameters *E*_*i*_(*i* = 1, 2) and it reduces for *E*_3_. The obtained results from Figs 26 and 27 show increasing behavior of heat transfer coefficient for larger values of *k* and Brinkman number *Br*.

### 0.4 Concentration

**Fig 28:**
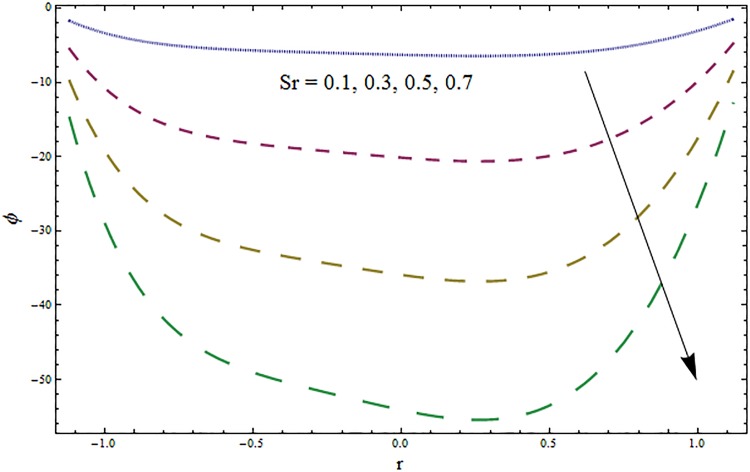
Variation in concentration *ϕ* for Soret number *Sr* when *ϵ* = 0.2, *t* = 0.1, *x* = 0.2, *k* = 5, *E*_1_ = 0.3, *E*_2_ = 0.2, *E*_3_ = 0.1, *β* = 0.5, *H* = 0.8, *Bi*_1_ = 10, *Sc* = 0.3, *Bi*_2_ = 10, *Du* = 15, *H* = 0.8, Pr = 2 and *Br* = 2.

**Fig 29:**
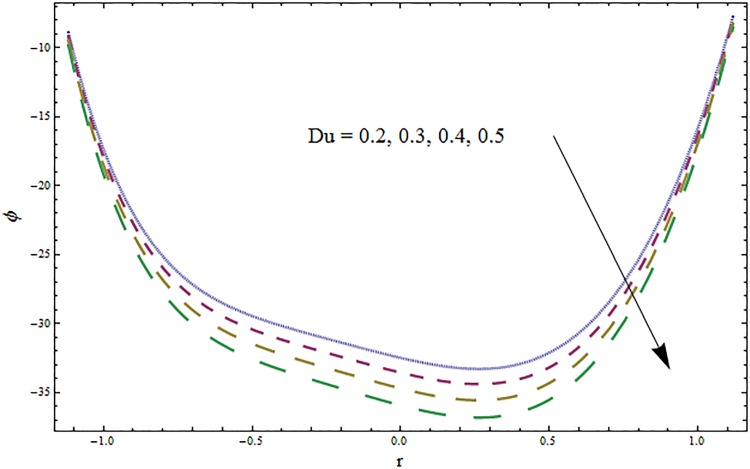
Variation in concentration *ϕ* for Dufour number *Du* when *ϵ* = 0.2, *t* = 0.1, *x* = 0.2, *k* = 5, *E*_1_ = 0.3, *E*_2_ = 0.2, *E*_3_ = 0.1, *β* = 0.5, *H* = 0.8, *Bi*_1_ = 10, *Sc* = 0.3, *Bi*_2_ = 10, *H* = 0.8, *Sr* = 5, Pr = 2 and *Br* = 2.

**Fig 30:**
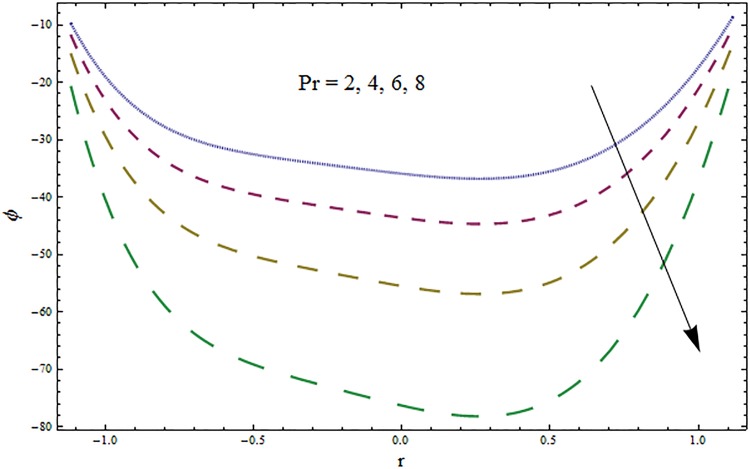
Variation in concentration *ϕ* for Prandtl number Pr when *ϵ* = 0.2, *t* = 0.1, *x* = 0.2, *k* = 5, *E*_1_ = 0.3, *E*_2_ = 0.2, *E*_3_ = 0.1, *Br* = 2, *β* = 0.5, *Bi*_1_ = 10, *Bi*_2_ = 10, *H* = 0.8, *Du* = 15, *Sr* = 5 and *Sc* = 0.3.

**Fig 31:**
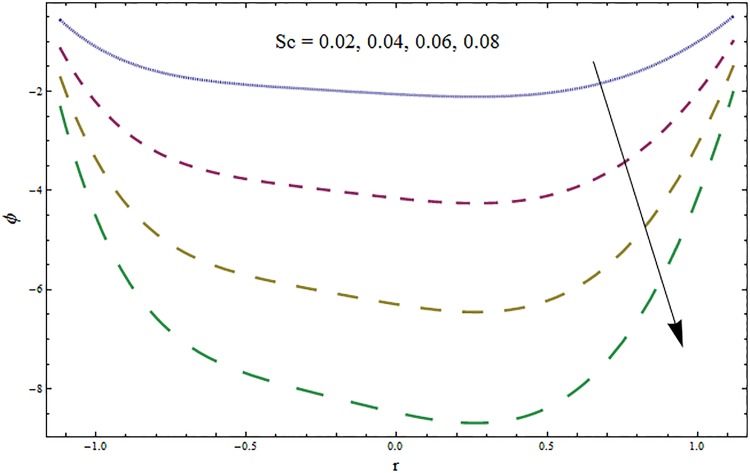
Variation in concentration *ϕ* for Schmidt number *Sc* when *ϵ* = 0.2, *t* = 0.1, *x* = 0.2, *k* = 5, *E*_1_ = 0.3, *E*_2_ = 0.2, *E*_3_ = 0.1, *Br* = 2, *β* = 0.5, *Bi*_1_ = 10, *Bi*_2_ = 10, *H* = 0.8, *Du* = 15, *Sr* = 5 and Pr = 2.

**Fig 32:**
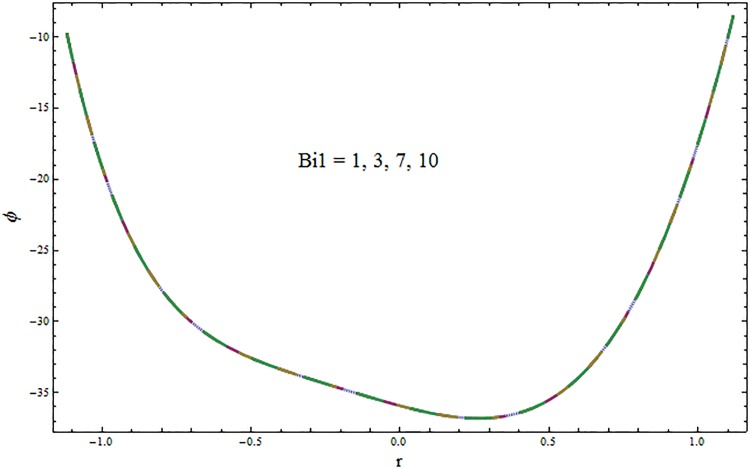
Variation in concentration *ϕ* for heat transfer Biot number *Bi*_1_ when *ϵ* = 0.2, *t* = 0.1, *x* = 0.2, *k* = 5, *E*_1_ = 0.3, *E*_2_ = 0.2, *E*_3_ = 0.1, *Br* = 2, *β* = 0.5, *Sc* = 0.3, *Bi*_2_ = 10, *H* = 0.8, *Du* = 15, *Sr* = 5 and Pr = 2.

**Fig 33:**
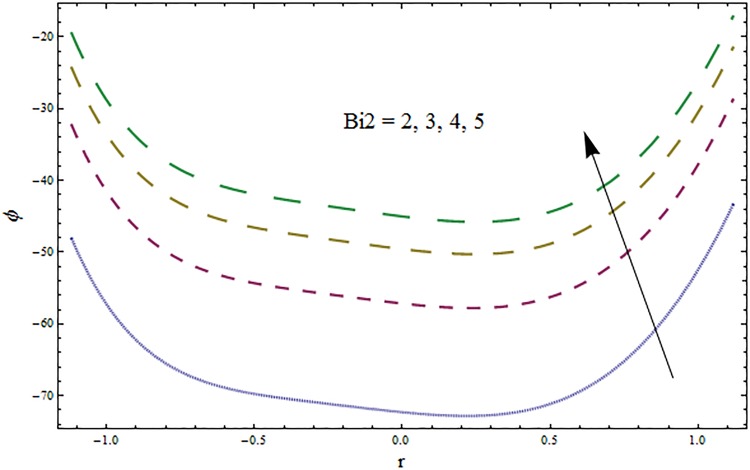
Variation in concentration *ϕ* for mass transfer Biot number *Bi*_2_ when *ϵ* = 0.2, *t* = 0.1, *x* = 0.2, *k* = 5, *E*_1_ = 0.3, *E*_2_ = 0.2, *E*_3_ = 0.1, *Br* = 2, *β* = 0.5, *Bi*_1_ = 10, *Sc* = 0.3, *H* = 0.8, *Du* = 15, *Sr* = 5 and Pr = 2.

**Fig 34:**
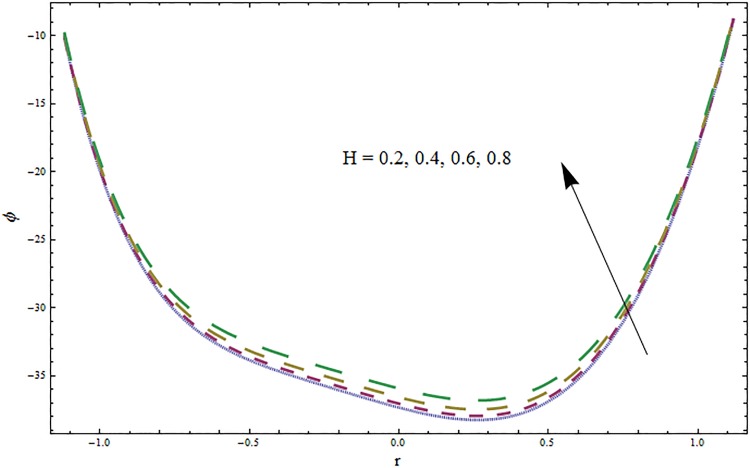
Variation in concentration *ϕ* for Hartmann number *H* when *ϵ* = 0.2, *t* = 0.1, *x* = 0.2, *k* = 5, *E*_1_ = 0.3, *E*_2_ = 0.2, *E*_3_ = 0.1, *Br* = 2, *β* = 0.5, *Bi*_1_ = 10, *Sc* = 0.3, *Bi*_2_ = 10, *Du* = 15, *Sr* = 5 and Pr = 2.

**Fig 35:**
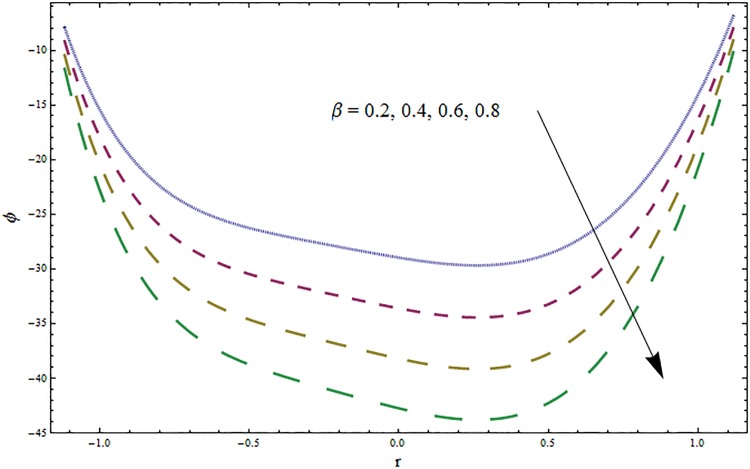
Variation in concentration *ϕ* for ratio of relaxation and retardation times parameter *β* when *ϵ* = 0.2, *t* = 0.1, *x* = 0.2, *k* = 5, *E*_1_ = 0.3, *E*_2_ = 0.2, *E*_3_ = 0.1, *Br* = 2, *H* = 0.8, *Bi*_1_ = 10, *Sc* = 0.3, *Bi*_2_ = 10, *Du* = 15, *Sr* = 5 and Pr = 2.

**Fig 36:**
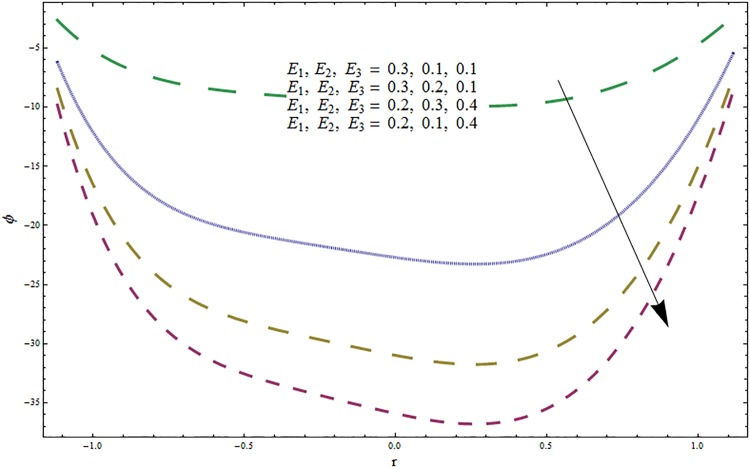
Variation in concentration *ϕ* for wall parameters *E*_1_, *E*_2_, *E*_3_ when *ϵ* = 0.2, *t* = 0.1, *x* = 0.2, *k* = 5, *H* = 0.8, *Br* = 2, *β* = 0.5, *Bi*_1_ = 10, *Sc* = 0.3, *Bi*_2_ = 10, *Du* = 15, *Sr* = 5 and Pr = 2.

**Fig 37:**
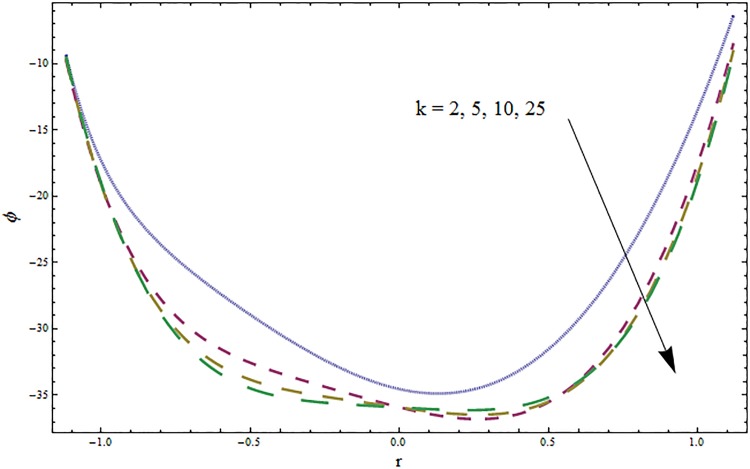
Variation in concentration *ϕ* for curvature parameter *k* when *ϵ* = 0.2, *t* = 0.1, *x* = 0.2, *β* = 0.5, *E*_1_ = 0.3, *E*_2_ = 0.2*E*_3_ = 0.1, *Br* = 2, *H* = 0.8, *Bi*_1_ = 10, *Sc* = 0.3, *Bi*_2_ = 10, *Du* = 15, *Sr* = 5 and Pr = 2.

**Fig 38:**
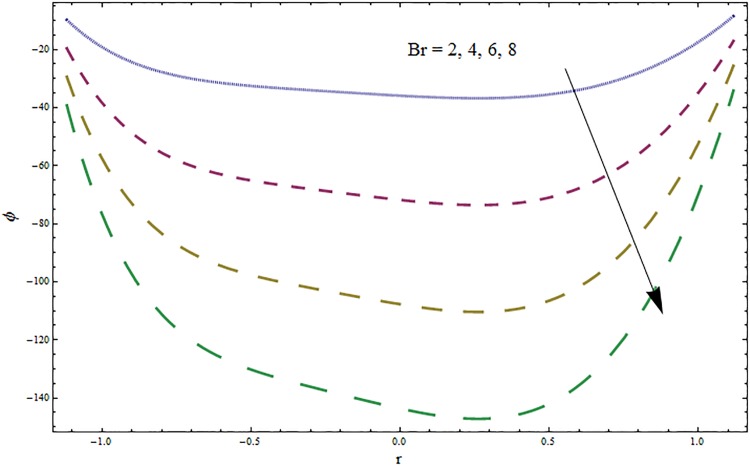
Variation in concentration *ϕ* for Brinkman number *Br* when *ϵ* = 0.2, *t* = 0.1, *x* = 0.2, *k* = 5, *E*_1_ = 0.3, *E*_2_ = 0.2, *E*_3_ = 0.1, *β* = 0.5, *H* = 0.8, *Bi*_1_ = 10, *Sc* = 0.3, *Bi*_2_ = 10, *Du* = 15, *Sr* = 5 and Pr = 2.

From Figs (28)–(38) the concentration profiles have been mentioned graphically. Opposite behaviour for concentration profile is noted when compared with temperature. The overall effect of concentration distribution is greater inside the walls of channel. In clinical practice the nutrients diffuse out of the blood vessels to neighboring tissues and thickens the mass of vessels. In Figs 28 and 29 the concentration decreases with the increase in Soret and Dufour numbers. Moreover the effect is also decreasing with the increase in Prandtl and Schmidt numbers (see Figs 30 and 31). By increasing Schmidt *Sc* number the mass diffusion decreases which show decrease in concentration. The heat transfer Biot number *Bi*_1_ has negligible effect on concentration distribution (see Fig 32). Increasing effect of mass transfer Biot number *Bi*_2_ on concentration distribution has been observed in Fig 33 when compared with *Bi*_1_. The obtained result from Fig 34 show that concentration enhances with increasing values of Hartmann number. In Fig 35 the concentration decreases for wall parameters which is opposite to temperature. Figs 36–38 show similar behavior of concentration distribution for increasing values of ratio of relaxation and retardation times parameter *β*, curvature parameter *k* and Brinkman number *Br*. It is because of the fact that the concentration distribution is higher when compared to straight channel in the upper half portion.

### 0.5 Streamlines pattern

**Fig 39:**
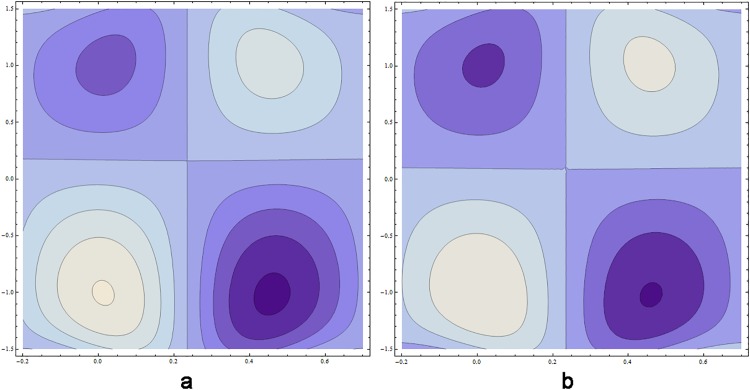
Streamlines for Hartmann number *H* when *E*_1_ = 0.3, *E*_2_ = 0.2, *E*_3_ = 0.3, *ϵ* = 0.1, *t* = 0, *β* = 0.4, *k* = 4, (a) *H* = 4.5 (b) *H* = 6.5.

**Fig 40:**
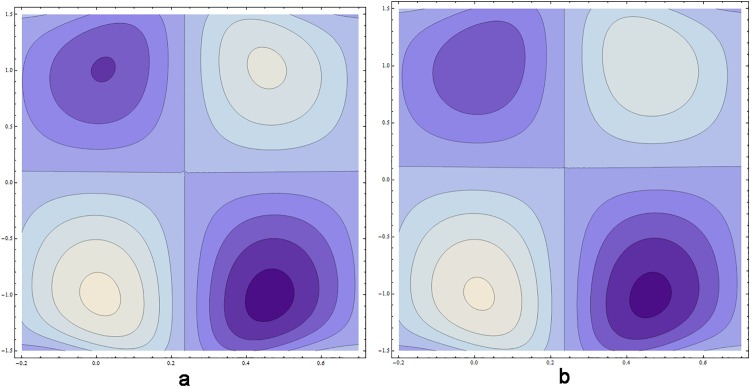
Streamlines for relaxation and retardation times parameter when *E*_1_ = 0.3, *E*_2_ = 0.2, *E*_3_ = 0.3, *ϵ* = 0.1, *t* = 0, *H* = 5, *k* = 4, (a) *β* = 0.4, (b) *β* = 1.

Fig 39(a) and 39(b) reveal that increasing values of Hartmann number *H* decrease the number of circulating streamlines in the lower half of the channel whereas size of bolus enhances. However the pattern of streamlines remain unchanged in the upper half of the channel. It is noticed from Fig 40(a) and 40(b) that larger values of *β* increases the number of circulating streamlines in the upper half of channel whereas size of bolus contracts.

## Concluding remarks

Peristaltic flow of Jeffrey fluid in curved channel with compliant walls is studied in the presence of convective boundary conditions and radial magnetic field effect. The major findings of presented analysis are listed below:

- Similar response of wall parameters on the velocity, temperature and heat transfer coefficient is noticed inside the curved channel.
- Temperature is increasing function of Brinkman, Soret, Dufour, Prandtl, and Schmidt numbers.
- Temperature decreases for larger heat and mass transfer Biot numbers.
- Concentration drops with the increase in Soret, Dufour, Prandtl, Schmidt and Brinkman numbers and it increases for heat and mass transfer Biot numbers.
- Opposite effects of Hartmann number and ratio of relaxation and retardation times parameter *β* are observed for the velocity, temperature, concentration and heat transfer coefficient.
- Similar effects of curvature parameter are seen on the velocity, temperature, concentration and heat transfer coefficient.
- Behavior of streamline pattern is increasing with ratio of relaxation and retardation times parameter.
- The Hartmann number on streamline pattern has opposite effects when compared with the ratio of relaxation and retardation times parameter.

### Appendix

$$\begin{array}{c} D_{1}=C_{1}\left(1+\sqrt{1+H^{2}\left(1+\beta \right)}\right),\;D_{2}=C_{2}\left(1-\sqrt{1+H^{2}\left(1+\beta \right)}\right),\;D_{3}=\frac{L}{H^{2}\left(1+\beta \right)},\;D_{4}=0, \end{array}$$

$$\begin{array}{c} C_{1}=-\frac{L(k{\left(k-\eta \right)}^{\sqrt{1+H^{2}+\beta H^{2}}}-{\left(k-\eta \right)}^{\sqrt{1+H^{2}+\beta H^{2}}}\eta -k{\left(k+\eta \right)}^{\sqrt{1+H^{2}+\beta H^{2}}}-{\left(k+\eta \right)}^{\sqrt{1+H^{2}+\beta H^{2}}}\eta )}{H^{2}\left(1+\beta \right)\left({\left(k-\eta \right)}^{2\sqrt{1+H^{2}+\beta H^{2}}}-{\left(k+\eta \right)}^{2\sqrt{1+H^{2}+\beta H^{2}}}\right)}, \end{array}$$

$$\begin{array}{c} C_{2}=-\frac{\begin{array}{c} L{\left(k-\eta \right)}^{\sqrt{1+H^{2}+\beta H^{2}}}{\left(k+\eta \right)}^{\sqrt{1+H^{2}+\beta H^{2}}}(k{\left(k-\eta \right)}^{\sqrt{1+H^{2}+\beta H^{2}}}+{\left(k-\eta \right)}^{\sqrt{1+H^{2}+\beta H^{2}}}\eta \\ -k{\left(k+\eta \right)}^{\sqrt{1+H^{2}+\beta H^{2}}}+{\left(k+\eta \right)}^{\sqrt{1+H^{2}+\beta H^{2}}}\eta ) \end{array}}{H^{2}\left(1+\beta \right)\left({\left(k-\eta \right)}^{2\sqrt{1+H^{2}+\beta H^{2}}}-{\left(k+\eta \right)}^{2\sqrt{1+H^{2}+\beta H^{2}}}\right)}, \end{array}$$

$$\begin{array}{ccc} C_{3} & = & Br{\left(4\left(-1+Du\mathrm{Pr}ScSr\right)\left(1+\beta \right){\left(1+H^{2}+\beta H^{2}\right)}^{\frac{3}{2}}\right)}^{-1}\\ & & (-2-2H^{2}-2H^{2}\beta +2\sqrt{1+H^{2}\left(1+\beta \right)}+H^{2}\sqrt{1+H^{2}\left(1+\beta \right)}+H^{2}\beta \sqrt{1+H^{2}\left(1+\beta \right)}),\\ C_{4} & = & Br{\left(4\left(-1+Du\mathrm{Pr}ScSr\right)\left(1+\beta \right){\left(1+H^{2}+\beta H^{2}\right)}^{\frac{3}{2}}\right)}^{-1}\\ & & (2+2H^{2}+2H^{2}\beta +2\sqrt{1+H^{2}\left(1+\beta \right)}+H^{2}\sqrt{1+H^{2}\left(1+\beta \right)}+H^{2}\beta \sqrt{1+H^{2}\left(1+\beta \right)}),\\ C_{5} & = & BrH^{2}{\left(-1+Du\mathrm{Pr}ScSr\right)}^{-1} \end{array}$$

$$\begin{array}{ccc} A_{1} & = & -\frac{1}{Bi_{1}\left(\frac{1}{k-\eta }+\frac{1}{k+\eta }-Bi_{1}Log\left[k-\eta \right]+Bi_{1}Log\left[k+\eta \right]\right)}\\ & & [a_{1}Bi_{1}Br\{\frac{1}{\left(1+\beta \right){\left(1+H^{2}\left(1+\beta \right)\right)}^{\frac{3}{2}}}(-Bi_{1}C_{1}^{2}(H^{2}\left(1+\beta \right)((-2+\sqrt{1+H^{2}\left(1+\beta \right)}\\ & & +2\left(-1+\sqrt{1+H^{2}\left(1+\beta \right)}\right)){\left(k-\eta \right)}^{2\sqrt{1+H^{2}+\beta H^{2}}})-Bi_{1}C_{2}^{2}(2\left(1+\sqrt{1+H^{2}\left(1+\beta \right)}\right)\\ & & +H^{2}\left(1+\beta \right)\left(2+\sqrt{1+H^{2}\left(1+\beta \right)}\right)){\left(k-\eta \right)}^{-2\sqrt{1+H^{2}+\beta H^{2}}})\\ & & +\frac{1}{\left(1+\beta \right)(1+H^{2}\left(1+\beta \right))}(-2C_{2}^{2}(2\left(1+\sqrt{1+H^{2}\left(1+\beta \right)}\right)\\ & & +H^{2}\left(1+\beta \right)\left(2+\sqrt{1+H^{2}\left(1+\beta \right)}\right)){\left(k-\eta \right)}^{-1-2\sqrt{1+H^{2}+\beta H^{2}}}\\ & & +2C_{1}^{2}\left(H^{2}\left(1+\beta \right)\left(\left(-2+\sqrt{1+H^{2}\left(1+\beta \right)}+2\left(-1+\sqrt{1+H^{2}\left(1+\beta \right)}\right)\right){\left(k-\eta \right)}^{-1+2\sqrt{1+H^{2}+\beta H^{2}}}\right)\right)\\ & & -\frac{8C_{1}C_{2}H^{2}Log\left[k-\eta \right]}{k-\eta }+4Bi_{1}C_{1}C_{2}H^{2}Log{\left[k-\eta \right]}^{2}\}\\ & & +Bi_{1}\{\frac{1}{4\left(-1+Du\mathrm{Pr}ScSr\right)\left(1+\beta \right){\left(1+H^{2}+\beta H^{2}\right)}^{\frac{3}{2}}}(Bi_{1}BrC_{2}^{2}(2\left(1+\sqrt{1+H^{2}\left(1+\beta \right)}\right)\\ & & +H^{2}\left(1+\beta \right)\left(2+\sqrt{1+H^{2}\left(1+\beta \right)}\right){\left(k+\eta \right)}^{-2\sqrt{1+H^{2}+\beta H^{2}}}+Bi_{1}BrC_{1}^{2}(2\left(-1+\sqrt{1+H^{2}\left(1+\beta \right)}\right)\\ & & +H^{2}\left(1+\beta \right)\left(-2+\sqrt{1+H^{2}\left(1+\beta \right)}\right){\left(k+\eta \right)}^{2\sqrt{1+H^{2}+\beta H^{2}}})\\ & & +\frac{1}{2\left(-1+Du\mathrm{Pr}ScSr\right)\left(1+\beta \right)\left(1+H^{2}+\beta H^{2}\right)}(-BrC_{2}^{2}(2\left(1+\sqrt{1+H^{2}\left(1+\beta \right)}\right)\\ & & +H^{2}\left(1+\beta \right)\left(2+\sqrt{1+H^{2}\left(1+\beta \right)}\right){\left(k+\eta \right)}^{-1-2\sqrt{1+H^{2}+\beta H^{2}}}+BrC_{1}^{2}(2\left(-1+\sqrt{1+H^{2}\left(1+\beta \right)}\right)\\ & & +H^{2}\left(1+\beta \right)\left(-2+\sqrt{1+H^{2}\left(1+\beta \right)}\right){\left(k+\eta \right)}^{-1+2\sqrt{1+H^{2}+\beta H^{2}}})\\ & & -\frac{2BrC_{1}C_{2}H^{2}Log\left[k+\eta \right]}{\left(-1+Du\mathrm{Pr}ScSr)(k+\eta \right)}+\frac{Bi_{1}BrC_{1}C_{2}H^{2}Log{\left[k+\eta \right]}^{2}}{1-Du\mathrm{Pr}ScSr}\}], \end{array}$$

$$\begin{array}{ccc} A_{2} & = & -\frac{1}{Bi_{1}}\{\frac{1}{4\left(-1+Du\mathrm{Pr}ScSr\right)\left(1+\beta \right){\left(1+H^{2}+\beta H^{2}\right)}^{\frac{3}{2}}}\\ & & (Bi_{1}BrC_{2}^{2}(2(H^{2}\left(1+\beta \right)(2+\sqrt{1+H^{2}\left(1+\beta \right)}\\ & & +\left(1+\sqrt{1+H^{2}\left(1+\beta \right)}\right)){\left(k+\eta \right)}^{-2\sqrt{1+H^{2}+\beta H^{2}}}\\ & & +Bi_{1}BrC_{1}^{2}(H^{2}\left(1+\beta \right)(-2+\sqrt{1+H^{2}\left(1+\beta \right)}\\ & & +2\left(-1+\sqrt{1+H^{2}\left(1+\beta \right)}\right)){\left(k+\eta \right)}^{2\sqrt{1+H^{2}+\beta H^{2}}})\\ & & +\frac{1}{2\left(-1+Du\mathrm{Pr}ScSr\right)\left(1+\beta \right)\left(1+H^{2}+\beta H^{2}\right)}(-BrC_{2}^{2}(2\left(1+\sqrt{1+H^{2}\left(1+\beta \right)}\right)\\ & & +H^{2}\left(1+\beta \right)\left(2+\sqrt{1+H^{2}\left(1+\beta \right)}\right){\left(k+\eta \right)}^{-1-2\sqrt{1+H^{2}+\beta H^{2}}}\\ & & +BrC_{1}^{2}\left(H^{2}\left(1+\beta \right)\left(-2++2\left(-1+\sqrt{1+H^{2}\left(1+\beta \right)}\right)\right){\left(k+\eta \right)}^{-1+2\sqrt{1+H^{2}+\beta H^{2}}}\right)\\ & & -\frac{2BrC_{1}C_{2}H^{2}Log\left[k+\eta \right]}{\left(-1+Du\mathrm{Pr}ScSr)(k+\eta \right)}+\frac{Bi_{1}BrC_{1}C_{2}H^{2}Log{\left[k+\eta \right]}^{2}}{1-Du\mathrm{Pr}ScSr}\}\\ & & +\frac{1}{Bi_{1}^{2}\left(\frac{1}{k-\eta }+\frac{1}{k+\eta }-Bi_{1}Log\left[k-\eta \right]+Bi_{1}Log\left[k+\eta \right]\right)}\left(\frac{1}{k+\eta }+Bi_{1}Log\left[k+\eta \right]\right)\\ & & a_{1}Bi_{1}Br\{\frac{1}{\left(1+\beta \right){\left(1+H^{2}\left(1+\beta \right)\right)}^{\frac{3}{2}}}(-Bi_{1}C_{1}^{2}(H^{2}\left(1+\beta \right)((-2+\sqrt{1+H^{2}\left(1+\beta \right)}\\ & & +2\left(-1+\sqrt{1+H^{2}\left(1+\beta \right)}\right)){\left(k-\eta \right)}^{2\sqrt{1+H^{2}+\beta H^{2}}}-Bi_{1}C_{2}^{2}(2\left(1+\sqrt{1+H^{2}\left(1+\beta \right)}\right)\\ & & +H^{2}\left(1+\beta \right)\left(2+\sqrt{1+H^{2}\left(1+\beta \right)}\right)){\left(k-\eta \right)}^{-2\sqrt{1+H^{2}+\beta H^{2}}})\\ & & +\frac{1}{\left(1+\beta \right)(1+H^{2}\left(1+\beta \right))}(-2C_{2}^{2}(2\left(1+\sqrt{1+H^{2}\left(1+\beta \right)}\right)\\ & & +H^{2}\left(1+\beta \right)\left(2+\sqrt{1+H^{2}\left(1+\beta \right)}\right)){\left(k-\eta \right)}^{-1-2\sqrt{1+H^{2}+\beta H^{2}}}\\ & & +2C_{1}^{2}(H^{2}\left(1+\beta \right)((-2+\sqrt{1+H^{2}\left(1+\beta \right)}\\ & & +2\left(-1+\sqrt{1+H^{2}\left(1+\beta \right)}\right)){\left(k-\eta \right)}^{-1+2\sqrt{1+H^{2}+\beta H^{2}}})\\ & & -\frac{8C_{1}C_{2}H^{2}Log\left[k-\eta \right]}{k-\eta }+4Bi_{1}C_{1}C_{2}H^{2}Log{\left[k-\eta \right]}^{2}\}\\ & & +Bi_{1}\{\frac{1}{4\left(-1+Du\mathrm{Pr}ScSr\right)\left(1+\beta \right){\left(1+H^{2}+\beta H^{2}\right)}^{\frac{3}{2}}}(Bi_{1}BrC_{2}^{2}(2\left(1+\sqrt{1+H^{2}\left(1+\beta \right)}\right)\\ & & +H^{2}\left(1+\beta \right)\left(2+\sqrt{1+H^{2}\left(1+\beta \right)}\right){\left(k+\eta \right)}^{-2\sqrt{1+H^{2}+\beta H^{2}}+Bi_{1}BrC_{1}^{2}(2\left(-1+\sqrt{1+H^{2}\left(1+\beta \right)}\right)}\\ & & +H^{2}\left(1+\beta \right)\left(-2+\sqrt{1+H^{2}\left(1+\beta \right)}\right){\left(k+\eta \right)}^{2\sqrt{1+H^{2}+\beta H^{2}}})\\ & & +\frac{1}{2\left(-1+Du\mathrm{Pr}ScSr\right)\left(1+\beta \right)\left(1+H^{2}+\beta H^{2}\right)}(-BrC_{2}^{2}(2\left(1+\sqrt{1+H^{2}\left(1+\beta \right)}\right)\\ & & +H^{2}\left(1+\beta \right)\left(2+\sqrt{1+H^{2}\left(1+\beta \right)}\right){\left(k+\eta \right)}^{-1-2\sqrt{1+H^{2}+\beta H^{2}}}+BrC_{1}^{2}(2\left(-1+\sqrt{1+H^{2}\left(1+\beta \right)}\right)\\ & & +H^{2}\left(1+\beta \right)\left(-2+\sqrt{1+H^{2}\left(1+\beta \right)}\right){\left(k+\eta \right)}^{-1+2\sqrt{1+H^{2}+\beta H^{2}}})\\ & & -\frac{2BrC_{1}C_{2}H^{2}Log\left[k+\eta \right]}{\left(-1+Du\mathrm{Pr}ScSr)(k+\eta \right)}+\frac{Bi_{1}BrC_{1}C_{2}H^{2}Log{\left[k+\eta \right]}^{2}}{1-Du\mathrm{Pr}ScSr}\}], \end{array}$$

$$\begin{array}{ccc} B_{1} & = & 2Bi_{2}kScSr-Bi_{2}^{2}k^{2}ScSrLog\left[k-\eta \right]+Bi_{2}^{2}\eta^{2}ScSrLog\left[k-\eta \right]\\ & & +Bi_{2}^{2}k^{2}ScSrLog\left[k+\eta \right]-Bi_{2}^{2}\eta^{2}ScSrLog\left[k+\eta \right],\\ B_{2} & = & Bi_{2}^{2}k^{2}ScSr\theta \left[-\eta \right]-Bi_{1}Bi_{2}k^{2}ScSr\theta \left[-\eta \right]+Bi_{1}Bi_{2}\eta^{2}ScSr\theta \left[-\eta \right]-Bi_{2}^{2}\eta^{2}ScSr\theta \left[-\eta \right]\\ & & +Bi_{1}Bi_{2}^{2}k^{2}ScSr\theta \left[-\eta \right]-Bi_{2}^{2}k^{2}ScSr\theta \left[-\eta \right]-Bi_{1}Bi_{2}\eta^{2}ScSr\theta \left[-\eta \right]+Bi_{2}^{2}\eta^{2}ScSr\theta \left[-\eta \right]\\ & & -Bi_{1}Bi_{2}\eta^{2}ScSr\theta \left[\eta \right]+Bi_{2}^{2}\eta^{2}ScSr\theta \left[\eta \right]+Bi_{1}Bi_{2}k^{2}ScSr\theta \left[\eta \right]-Bi_{2}^{2}k^{2}ScSr\theta \left[\eta \right],\\ B_{3} & = & Bi_{1}kScSr\theta \left[-\eta \right]-Bi_{2}kScSr\theta \left[-\eta \right]-Bi_{1}ScSr\eta \theta \left[-\eta \right]+Bi_{2}ScSr\eta \theta \left[-\eta \right]\\ & & +Bi_{1}kScSr\theta \left[\eta \right]-Bi_{2}kScSr\theta \left[\eta \right]+Bi_{1}\eta ScSr\theta \left[\eta \right]-Bi_{2}\eta ScSr\theta \left[\eta \right]\\ & & -Bi_{1}Bi_{2}k^{2}ScSrLog\left[k-\eta \right]\theta \left[\eta \right]+Bi_{2}^{2}k^{2}ScSrLog\left[k-\eta \right]\theta \left[\eta \right]+Bi_{1}Bi_{2}\eta^{2}ScSrLog\left[k-\eta \right]\theta \left[\eta \right]\\ & & -Bi_{2}^{2}\eta^{2}ScSrLog\left[k-\eta \right]\theta \left[\eta \right])/(Bi_{2}\left(-2k+Bi_{2}k^{2}Log\left[k-\eta \right]-Bi_{2}k^{2}Log\left[k+\eta \right]+Bi_{2}\eta^{2}Log\left[k+\eta \right]\right), \end{array}$$

$$\begin{array}{ccc} Z & = & \eta_{x}[\frac{A_{1}}{k+\eta }+\frac{1}{2\left(-1+Du\mathrm{Pr}ScSr\right)\left(1+\beta \right)\left(1+H^{2}+\beta H^{2}\right)}\\ & & \{BrC_{1}^{2}\left(-2-2H^{2}-2H^{2}\beta +2\sqrt{1+H^{2}\left(1+\beta \right)}\right)\\ & & +H^{2}\sqrt{1+H^{2}\left(1+\beta \right)}+H^{2}\beta \sqrt{1+H^{2}\left(1+\beta \right)}){\left(k+\eta \right)}^{-1+2\sqrt{1+H^{2}+\beta H^{2}}}\}\\ & & -\frac{1}{2\left(-1+Du\mathrm{Pr}ScSr\right)\left(1+\beta \right)\left(1+H^{2}+\beta H^{2}\right)}\{BrC_{2}^{2}(2+2H^{2}+2H^{2}\beta \\ & & +2\sqrt{1+H^{2}\left(1+\beta \right)})+H^{2}\sqrt{1+H^{2}\left(1+\beta \right)}+H^{2}\beta \sqrt{1+H^{2}\left(1+\beta \right)}){\left(k+\eta \right)}^{-1-2\sqrt{1+H^{2}+\beta H^{2}}}\}\\ & & -\frac{2BrC_{1}C_{2}H^{2}Log\left[k+\eta \right]}{\left(-1+Du\mathrm{Pr}ScSr)(k+\eta \right)}], \end{array}$$

$$\begin{array}{c} a_{1}=\left(1/4\left(-1+Du\mathrm{Pr}ScSr\right)\right). \end{array}$$
